# Polo-like kinase-1 mediates hepatitis C virus-induced cell migration, a drug target for liver cancer

**DOI:** 10.26508/lsa.202201630

**Published:** 2023-08-30

**Authors:** Korri E El-Khobar, Enoch Tay, Eve Diefenbach, Brian S Gloss, Jacob George, Mark W Douglas

**Affiliations:** 1 Storr Liver Centre, Westmead Institute for Medical Research, University of Sydney at Westmead Hospital, Westmead, Australia; 2 Eijkman Institute for Molecular Biology, Jakarta, Indonesia; 3 Protein Core Facility, Westmead Institute for Medical Research , Westmead, Australia; 4 Centre for Infectious Diseases and Microbiology, Sydney Infectious Diseases Institute, University of Sydney at Westmead Hospital, Westmead, Australia; 5 Westmead Research Hub, Westmead Institute for Medical Research , Westmead, Australia

## Abstract

Polo-like kinase 1 (PLK1) is a regulator of cell mitosis and cytoskeletal dynamics. *PLK1* overexpression in liver cancer is associated with tumour progression, metastasis, and vascular invasion. Hepatitis C virus (HCV) NS5A protein stimulates PLK1-mediated phosphorylation of host proteins, so we hypothesised that HCV–PLK1 interactions might be a mechanism for HCV-induced liver cancer. We used a HCV cell-culture model (Jc1) to investigate the effects of virus infection on the cytoskeleton. In HCV-infected cells, a novel posttranslational modification in β-actin was observed with phosphorylation at Ser239. Using in silico and in vitro approaches, we identified PLK1 as the mediating kinase. In functional experiments with a phosphomimetic mutant form of β-actin, Ser239 phosphorylation influences β-actin polymerization and distribution, resulting in increased cell motility. The changes were prevented by treating cells with the PLK1 inhibitor volasertib. In HCV-infected hepatocytes, increased cell motility contributes to cancer cell migration, invasion, and metastasis. PLK1 is an important mediator of these effects and early treatment with PLK1 inhibitors may prevent or reduce HCC progression, particularly in people with HCV-induced HCC.

## Abstract (precis)

This study identifies PLK1 as a novel protein that promotes liver cancer in people with hepatitis C. The findings suggest a novel drug target for patients with liver cancer to reduce cancer spread and improve survival.

## Introduction

Primary liver cancer (hepatocellular carcinoma [HCC]) is the second leading cause of cancer-related deaths worldwide (1). The prognosis for this cancer is poor, with curative strategies feasible only in a minority of cases (10–20%) (2). Hepatitis C virus (HCV) infection increases HCC risk by 15–20-fold (3) because of a combination of chronic inflammation, oxidative stress (4), and a range of virus-specific factors (5). Despite the success of direct-acting antivirals, the increased risk of liver cancer persists for over 10 yr after HCV cure in patients with F3 fibrosis or cirrhosis (6, 7). This persisting risk is not because of viral integration as unlike hepatitis B virus (HBV), the HCV RNA genome does not integrate into hepatocyte DNA (8). However, in patients with HCV-induced cirrhosis, monoclonal liver nodules are present which harbour premalignant changes that persist after HCV cure (9), thereby maintaining the increased risk of HCC.

HCC risk is two to three times higher in patients with cirrhosis because of HCV than in those with cirrhosis from other causes such as alcohol or metabolic (dysfunction)-associated fatty liver disease (MAFLD) (10). The virus has been shown to induce a range of pro-carcinogenic changes including effects on cell pathways that regulate cell survival, suppression of anti-tumour immunity, activation of oncogenes, induction of reactive oxidative stress, epigenetic changes, and promotion of epithelial–mesenchymal transition (EMT) (5, 11).

Polo-like kinase 1 (PLK1) is a key regulator of mitosis and cytoskeletal dynamics that is overexpressed in a range of tumours and is associated with tumour progression, metastasis, and vascular invasion (12, 13). In HCC, PLK1 is overexpressed and elevated PLK1 levels correlate with reduced survival, tumour thrombus, metastasis, clinical stage, tumour stage, and histological grade (14, 15, 16, 17).

In this context, the HCV NS5A protein interacts with PLK1 to promote viral replication (18). We hypothesised that interactions between HCV and PLK1 might be a mechanism for HCV-induced HCC invasion and metastasis. We used cell-culture models to examine HCV-induced activation of PLK1 and downstream effects on cell morphology, cytoskeletal proteins, and cell migration. We examined whether targeting PLK1 could reverse these changes, supporting a role for PLK1 inhibitors such as volasertib to treat patients with HCC.

## Results

### PLK1 expression is increased in HCC and correlates with metastatic disease

**Figure 1.**
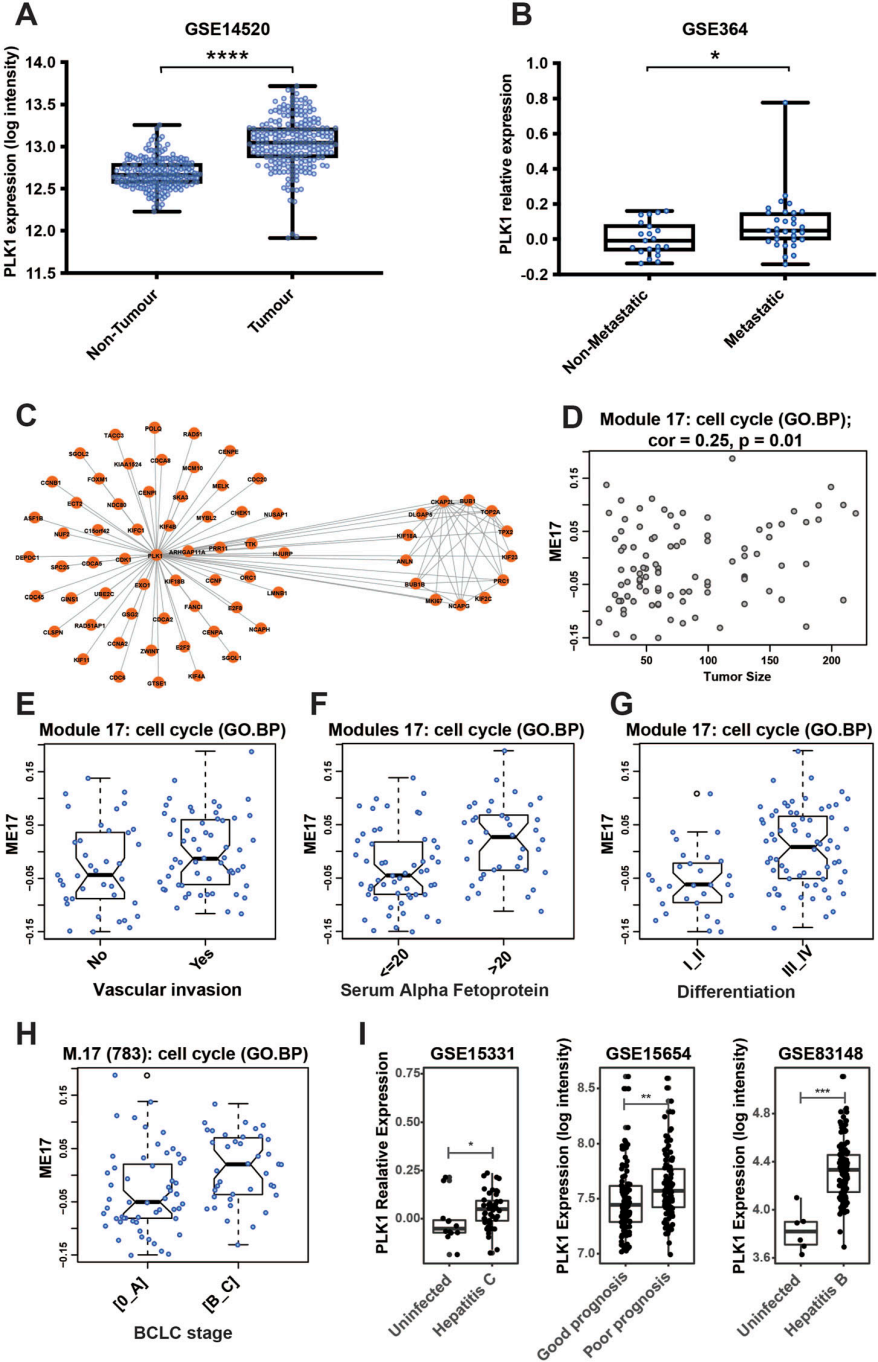
GEO dataset analyses of increased polo-like kinase 1 (*PLK1*) expression in hepatocellular carcinoma (HCC) tissues and its correlation with metastatic progression. **(A)** *PLK1* expression in HCC tissues was analysed using the GEO dataset of matched liver tumour and non-tumour tissue from 255 HCC patients (GSE14520). *PLK1* (probe 201429_s_at) was significantly increased in tumour tissue compared with non-tumour tissue. **(B)** The correlation of increased *PLK1* expression and HCC metastatic progression was analysed using the GEO dataset of HCC tumour tissues from 10 patients with and 10 patients without metastatic disease (GSE364). *PLK1* expression was higher in tumour tissues from patients with metastatic disease than in tumour tissues from patients with non-metastatic HCC (*P < 0.05; ****P < 0.0001). **(C)** Weighted gene co-expression network analysis of a HCC dataset (LICA-FR, 160 HCC cases). *PLK1* mapped to co-expression module M17 (cell cycle). Points are actual expression values (one dot per sample). **(D)** Expression of module eigengenes correlated with tumour size (*P* = 0.01). **(E, F, G, H)** Increased expression of M17 was associated with (E) more vascular invasion, (F) higher serum alpha-fetoprotein, (G) poorer tumour differentiation grade, and (H) more advanced BCLC stage. **(I)** Expression of *PLK1* in liver biopsy tissue from 30 people with chronic hepatitis C and 30 uninfected controls (GSE15331); patients with hepatitis C and early (Child–Pugh A) cirrhosis, comparing those with good prognosis and poor prognosis (GSE15654); 122 patients with chronic hepatitis B and uninfected controls (*P < 0.05; **P < 0.01; ***P < 0.001).

*PLK1* expression was analysed in a cohort of 225 patients with HCC (Gene expression omnibus [GEO] dataset GSE14520) (19). As shown in Fig 1A, *PLK1* expression was increased in tumour tissue compared with matched non-tumour liver (*P* < 0.0001). Next, PLK1 expression was measured in HCC from 10 patients with metastatic disease and 10 without (GEO dataset GSE364) (20). As shown in Fig 1B, *PLK1* expression was higher in tumours from patients with metastases than from those with localised diseases (*P* < 0.05).

We then performed weighted gene co-expression network analysis (21) using a French cancer dataset (ICGC, LICA-FR) containing RNA-seq data from 160 HCC cases, as recently published by our group (22). As shown in Fig 1C, *PLK1* mapped to co-expression module M17 that is involved in cell cycle regulation. This module was positively correlated with tumour size (shown in Fig 1D, cor = 0.25, *P* = 0.01). Higher expression level of M17 (cell cycle) was associated with increased vascular invasion, higher serum alpha-fetoprotein, and more advanced tumour grade and Barcelona Clinic Liver Cancer stage (shown in Fig 1E–H).

Finally, we looked for the effects of viral hepatitis versus nonviral liver disease on *PLK1* expression in the liver. As shown in Fig 1I, *PLK1* expression is increased in liver tissues from people with chronic hepatitis C compared with an uninfected liver (*P* < 0.05) (GEO dataset GSE15331) (23). In patients with hepatitis C and Child-Pugh A cirrhosis, *PLK1* expression was higher in patients with poor prognosis (*P* < 0.01) and predicts death, progression to advanced cirrhosis, and development of HCC over 10 yr (GSE15654) (24). Similar to hepatitis C, patients with chronic hepatitis B have higher *PLK1* expression in their livers than uninfected patients (GSE83148) (25). We performed similar analyses for patients with MAFLD but found no significant change in *PLK1* expression compared with patients without MAFLD (data not shown) (GSE66676, E-MEXP-3291) (26, 27).

In sum, *PLK1* expression is increased in HCC, particularly in the context of tumour metastasis and vascular invasion, suggesting a role for PLK1 in HCC cell motility. *PLK1* expression is increased in patients with hepatitis B or hepatitis C, but not in patients with MAFLD, suggesting a virus-specific effect that may contribute to the development and progression of HCC in patients with viral hepatitis.

### HCV induces posttranslational modification of β-actin, altering its cellular distribution

**Figure S1.**
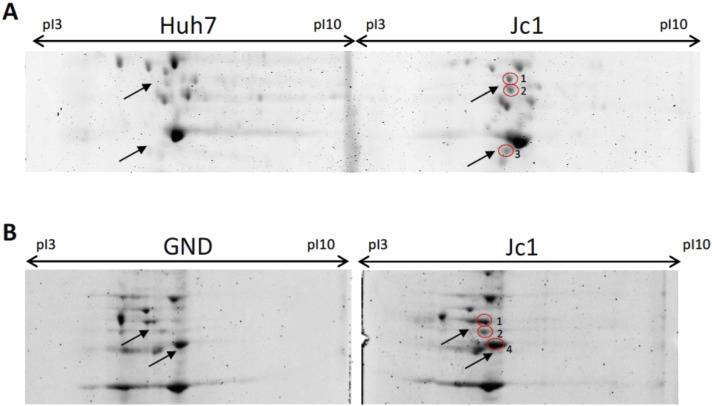
HCV increases α-tubulin, vimentin, and keratin expression. **(A, B)** 2-D gel electrophoresis (2-DE) analysis of solubilised membrane protein extracts from (A) replication-competent HCV (Jc1 strain) and uninfected Huh7 cells, and (B) replication-deficient HCV (GND-mutant) and HCV (Jc1 strain) cells. In Jc1-infected cells, there were increased (1) vimentin, (2) a-tubulin, (3) keratin type I (3), and (4) keratin type II (all encircled in red, marked by arrows), as identified by MALDI TOF-MS. The images shown are representative 2-DE gels from three independent experiments.

**Figure 2.**
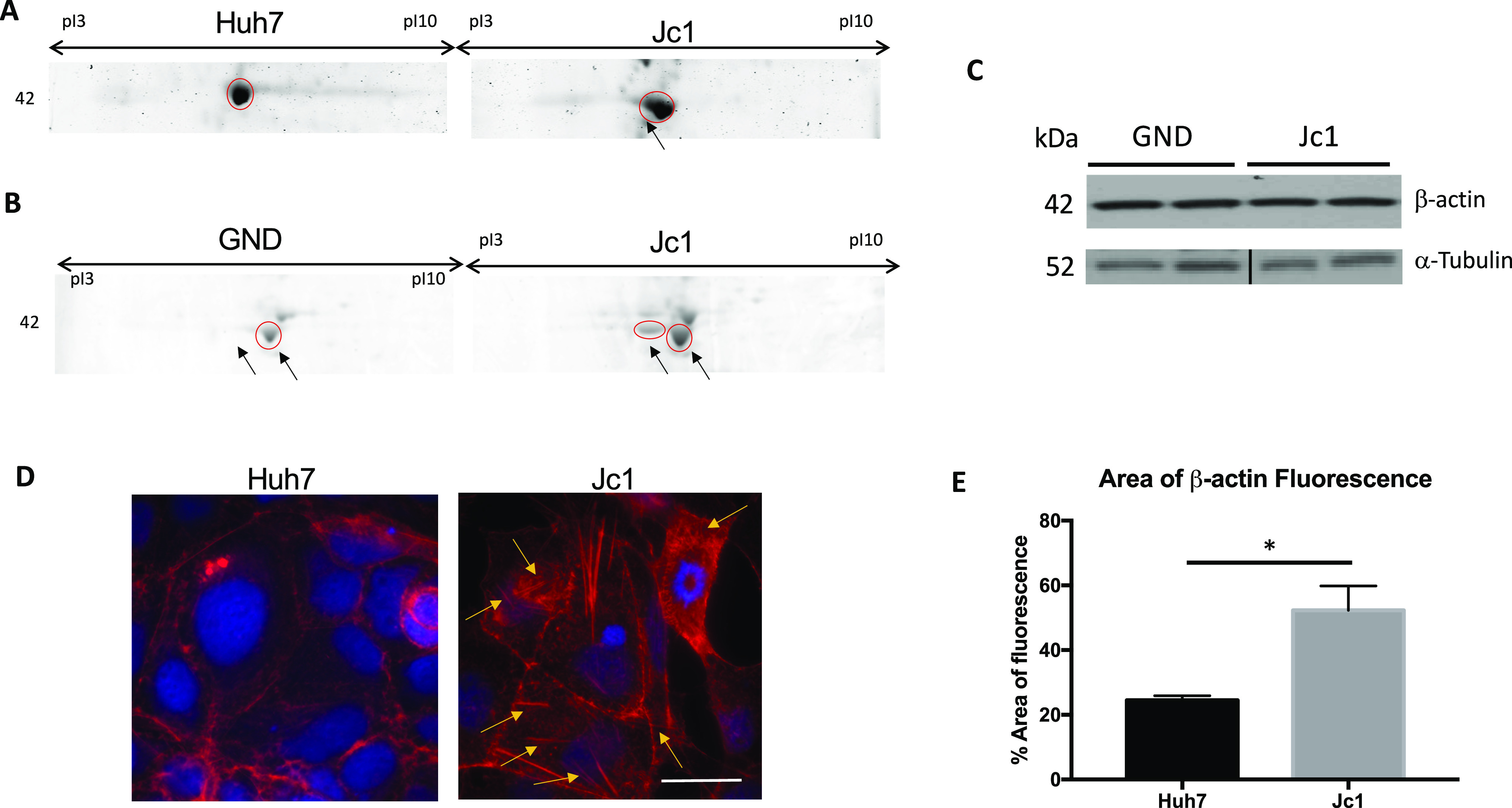
HCV induces posttranslational modification of β-actin and alteration of its cellular distribution. **(A, B)** 2-D gel electrophoresis (2-DE) analysis of solubilised membrane protein extracts from (A) replication competent HCV (Jc1 strain) and uninfected Huh7 cells, and (B) replication-deficient HCV (GND-mutant) and HCV (Jc1) cells. In Jc1-infected cells, there was an additional ∼40-kD-size protein spot (encircled in red, marked by arrows) identified by MALDI TOF-MS as β-actin. The images shown are representative 2-DE gels from three independent experiments. **(C)** β-actin and α-tubulin protein levels in cells infected with replication competent (Jc1) or replication-deficient (GND) HCV. A representative Western blot is shown. The black line in the tubulin blot indicates a splice in the blot to present only the GND and Jc1 bands. **(D)** Increased β-actin filament distribution (denoted by yellow arrows) in Jc1 infected cells compared with uninfected controls. Scale bar represents 15 μm. **(E)** Measurement of total area of β-actin fluorescence using FIJI. Each column represents the mean measurement from 20 cells in three different experiments. Error bar represent SEM (*P < 0.05). Source data are available for this figure.

**Figure S2.**
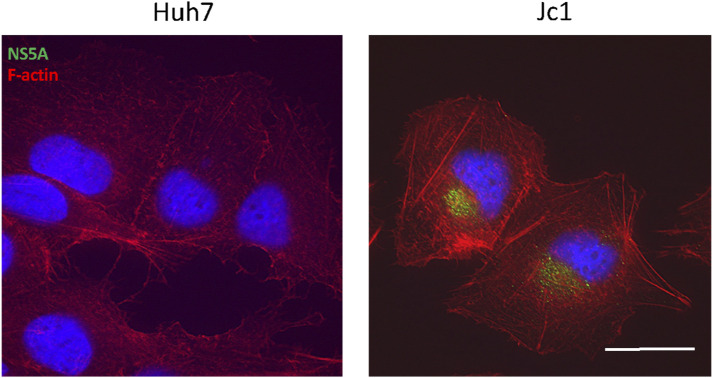
F-actin staining in HCV-infected cells. Expression of HCV NS5A (green) and F-actin (red, stained with phalloidin) in uninfected and HCV (Jc1) infected Huh7 cells. Nuclei were stained with DAPI (blue). Fluorescence signals were deconvolved using Softworx software. Representative images are shown. More than 20 cells were analysed. Scale bar represents 25 μm.

Proteomics analysis of membrane fractions from Huh7 cells infected with HCV (Jc1 strain) was performed using two-dimensional gel electrophoresis (2-DE) and matrix-assisted laser desorption ionization-time of flight mass spectrometry (MALDI TOF-MS). In HCV-infected cells, changes in several cytoskeletal proteins were observed, including α-tubulin, keratin, and vimentin (Fig S1). The most prominent difference was a wider, irregular protein spot in infected cells that was identified as β-actin (Fig 2A). This spot was only present in cells infected with replication competent HCV (Jc1), not a replication-deficient strain (GND) (Fig 2B), suggesting active viral replication was required. There was no difference in total β-actin or α-tubulin (Fig 2C), suggesting the change was because of posttranslational modification. Immunofluorescence microscopy showed that HCV (Jc1)-infected cells had more actin filaments (Fig 2D) and a greater area of β-actin (red) fluorescence (52.29% ± 7.52 versus 24.53% ± 1.37, *P* < 0.05; Fig 2E), suggesting posttranslational modification of β-actin alters actin dynamics. Similar results were observed in actin filaments staining with phalloidin (Fig S2).

### HCV induces EMT

**Figure 3.**
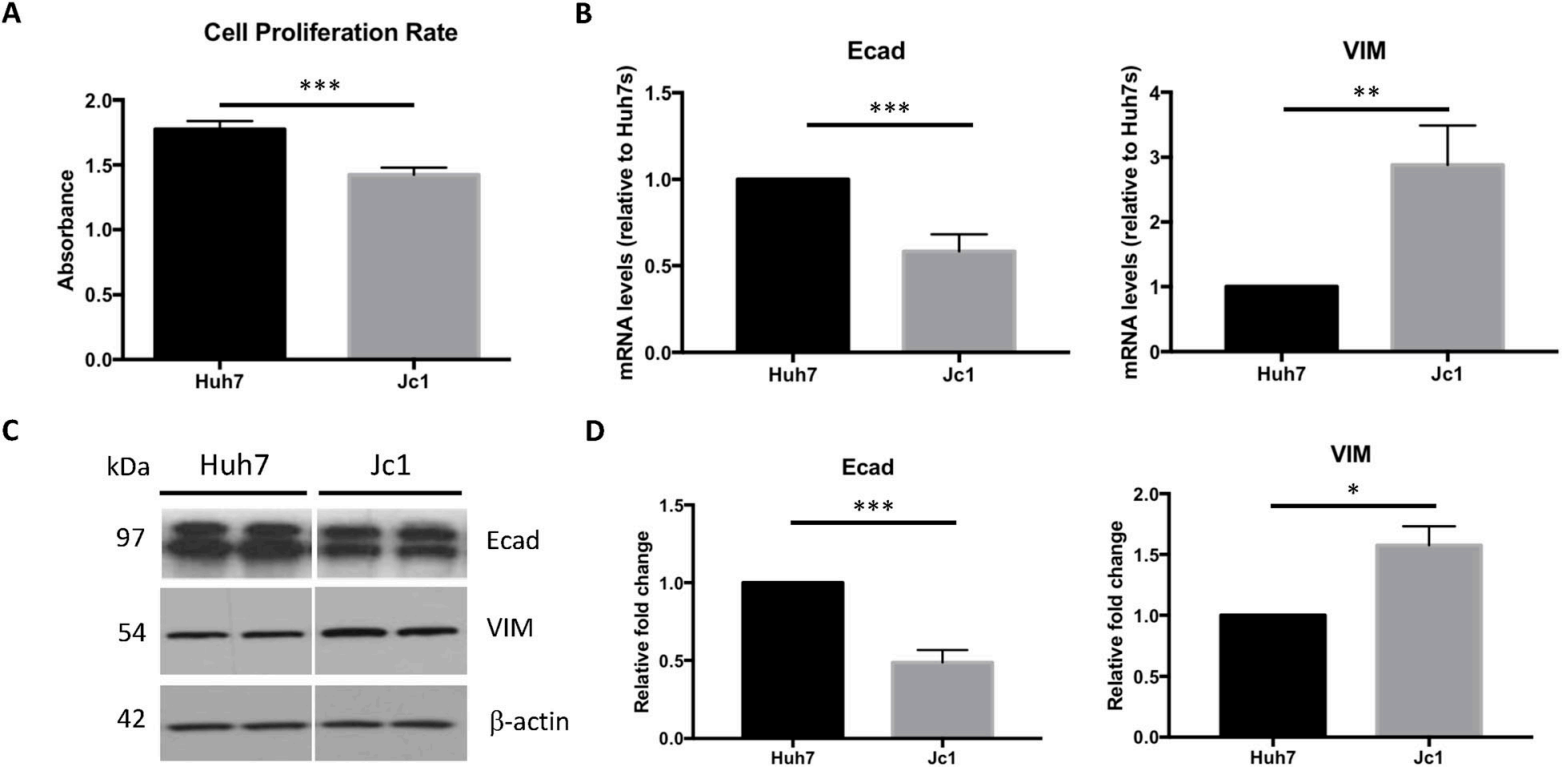
Changes in cell proliferation rate and epithelial–mesenchymal transition (EMT) markers expression in HCV-infected cells. **(A)** Measurement of cell proliferation rate was performed using Cell Proliferation BrdU ELISA kit. Absorbance value at 450 nm (OD450) correlates with the cell proliferation rate. **(B)** mRNA levels for E-cadherin (Ecad) and vimentin in HCV (Jc1)-infected cells. Huh7 mRNA levels were normalized to 1. **(C)** Protein levels of E-cadherin and vimentin in HCV (Jc1)-infected cells. A representative Western blot is shown. **(D)** Densitometry analyses of Western blots. Changes in protein expression are plotted relative to Huh7 controls. Each bar represents an average of three experiments and error bars represent SEM (*P < 0.05; **P < 0.01; ***P < 0.001).

HCV infection reduced cell proliferation, as measured by BrdU ELISA (1.42 ± 0.05 versus 1.78 ± 0.06, *P* < 0.001; Fig 3A). Consistent with HCV inducing EMT, the expression of E-cadherin (an epithelial marker) was reduced ∼50% (relative expression 0.58 ± 0.09, *P* < 0.001) and vimentin (a mesenchymal marker) increased threefold (2.88 ± 0.61, *P* < 0.01; shown in Fig 3B). Western blot (Fig 3C) confirmed reduced E-cadherin protein (0.49 ± 0.08, *P* < 0.001) and increased vimentin protein (1.58 ± 0.16, *P* < 0.05; Fig 3D).

### HCV-induced β-actin phosphorylation at serine 239 (S239) is mediated by PLK1

**Figure 4.**
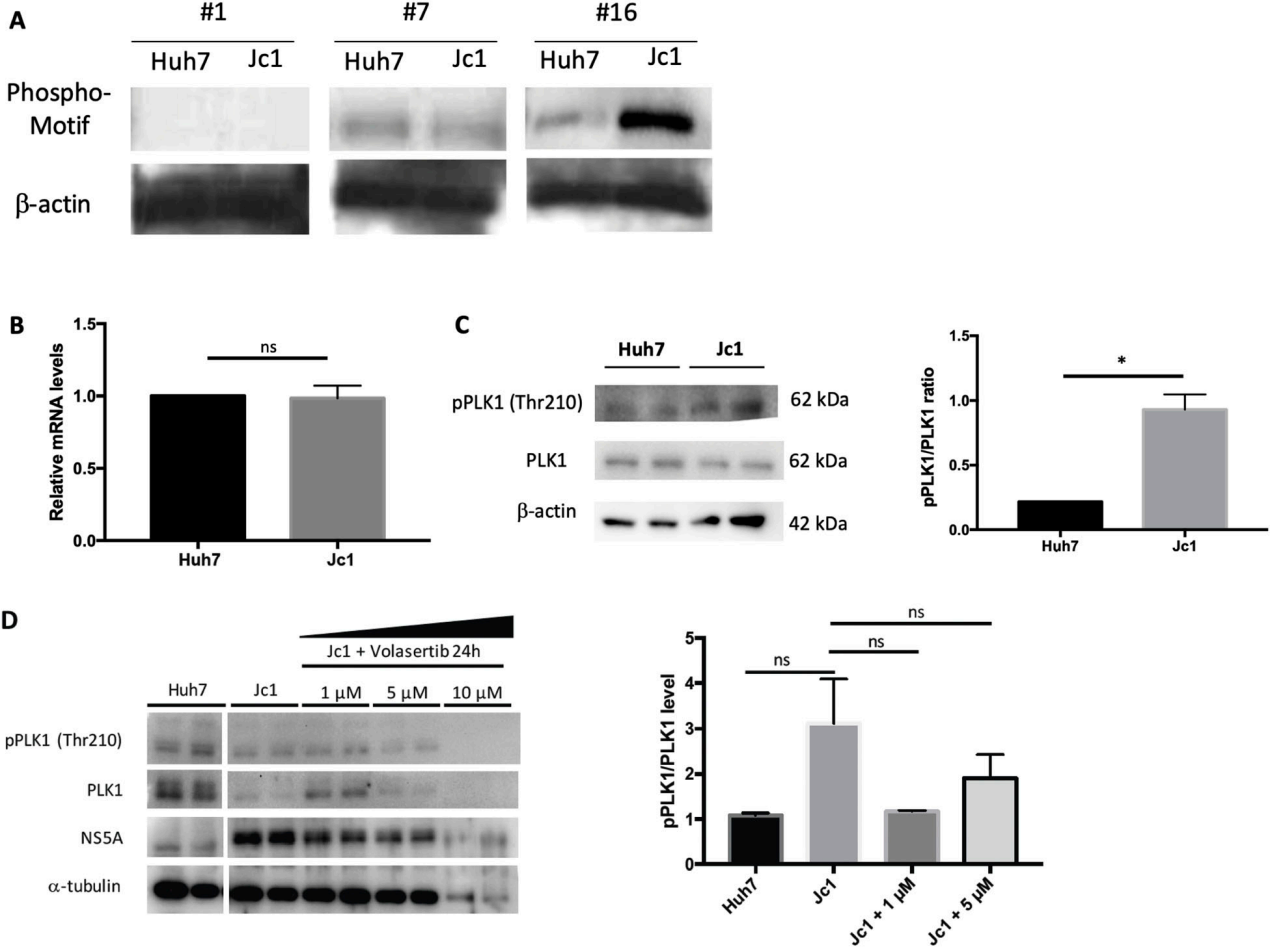
Polo-like kinase-1 mediates β-actin S239 phosphorylation. **(A)** Detection of β-actin phosphorylation sites using the KinomeView Profiling kit. β-actin was precipitated from HCV (Jc1)-infected and -uninfected Huh7 cell lysates and labelled with the 16 phosphomotif antibodies. Bands were detected only for antibodies #16 ((K/H)S*P motif) and #7 ((F/K)XX(F/Y)(S*/T*)(F/Y) motif), with none against the remaining 14 phosphomotifs. **(B)** PLK1 mRNA expression in HCV (Jc1)-infected cells relative to uninfected controls. mRNA levels of control were normalized to 1. **(C)** PLK1 protein expression in HCV-infected cells compared with control. Graph on the right panel showed PLK1 activation level (pPLK1/PLK1 ratios). **(D)** Reduced PLK1 and NS5A expression in HCV-infected cells after volasertib treatment. Uninfected and HCV-infected cells were treated with increasing doses of volasertib (1, 5, or 10 μM) and harvested 24 h after treatment. α-tubulin was used as a loading control. Graph on the right panel showed PLK1-activation levels (pPLK1/PLK1 ratios) in uninfected, HCV-infected, and HCV-infected cells treated with volasertib (*P < 0.05).

S239 was identified as the most likely site of β-actin phosphorylation using NetPhos 3.1 Server software, with a high confidence score of 0.989. Consistent with this, phosphorylation of β-actin S239 was detected in lysates from HCV (Jc1)-infected cells using the KinomeView Profiling kit, with a specific band detected by antibody #16 against the (K/H)S*P phosphomotif (Fig 4A).

**Figure S3.**
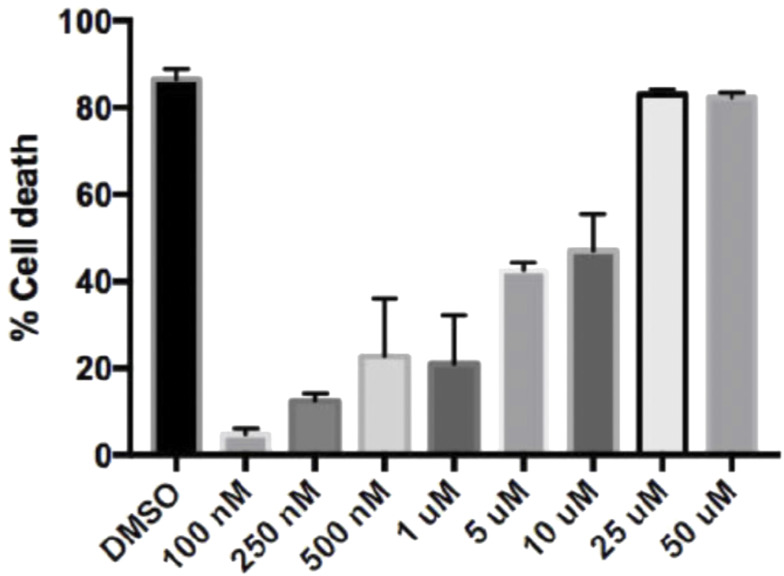
Effect of volasertib treatment on Huh7 cell death. Huh7 cells were treated with increasing doses of volasertib, ranging from 100 nM to 50 μM for 24 h. The viable cell percentage on treated Huh7s was determined by comparing the average OD488 values of treated cells with non-treated cells. The percentage of cell death was calculated by subtracting the viable cell percentage from 100%. Each bar represents an average of three experiments and error bars represent SEM.

**Figure S4.**
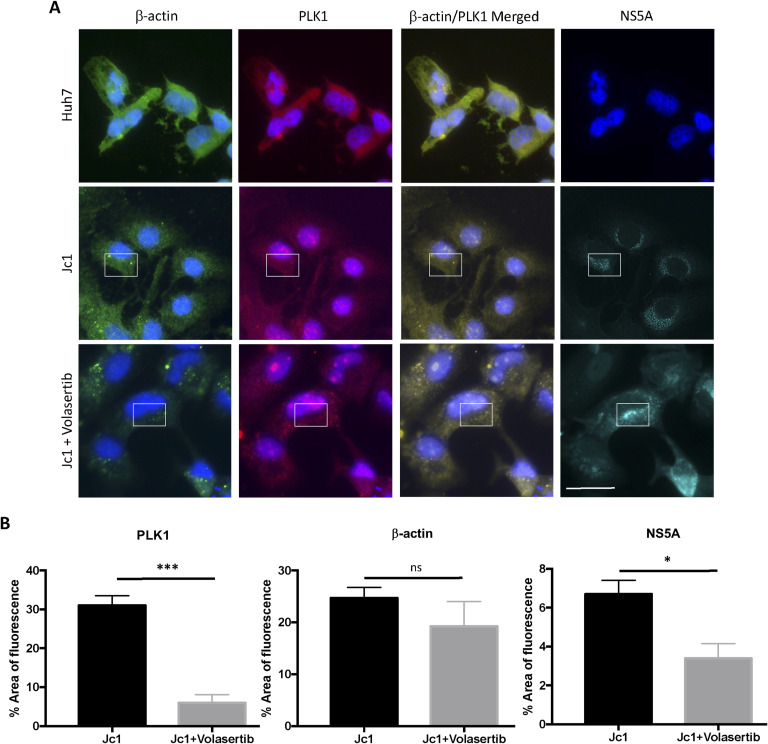
Effect of volasertib treatment on actin in HCV-infected Huh7 cells. HCV-infected Huh7 (Jc1) cells were treated with an optimal dose of volasertib for 24 h. **(A)** Expression of β-actin (green), PLK1 (red), and HCV NS5A (cyan) in Huh7 controls (top panel), Jc1-infected cells (middle panel), and volasertib-treated Jc1-infected cells (bottom panel). Nuclei were stained with DAPI (blue). β-actin, PLK1, and NS5A seem to localise in the same region near the nucleus (area within the white box). Representative images are shown. More than 20 cells were analysed. Scale bar represents 25 μm. **(B)** Measurement of total area of PLK1, β-actin, and NS5A fluorescence using FIJI software. Each column represents the mean measurement from 20 cells in three different experiments. Error bar represents SEM (*P < 0.05; **P < 0.01; ***P < 0.001; ns, not significant).

PLK was predicted to be the kinase responsible for phosphorylating β-actin S239, using NetPhosK 1.0 Server and Group-based Prediction System 3.0 software. There was no significant difference in *PLK1* mRNA (Fig 4B) or total PLK1 protein in HCV (Jc1)-infected cells, but there was a threefold increase in PLK1 activation (phospho-PLK1/PLK1 ratio) (0.93 ± 0.11 versus 0.22 ± 0.01, *P* < 0.05; Fig 4C). HCV-infected cells were then treated with the PLK1 inhibitor, volasertib (BI6727). There was a dose-dependent reduction in phospho-PLK1 and total PLK1, with a nonsignificant trend towards reduced PLK1 activation (pPLK1/PLK1) (shown in Fig 4D). The volasertib concentration required to inhibit growth by 50% (IC50) in our model was 6.28 μM (Fig S3), so this dose was chosen for subsequent experiments. HCV-infected Huh7 cells treated with volasertib appeared to have less actin compared with untreated cells (Fig S4), but there was no significant difference in the area of actin staining.

### PLK1 interacts with the actin cytoskeleton in HCV-infected cells

**Figure 5.**
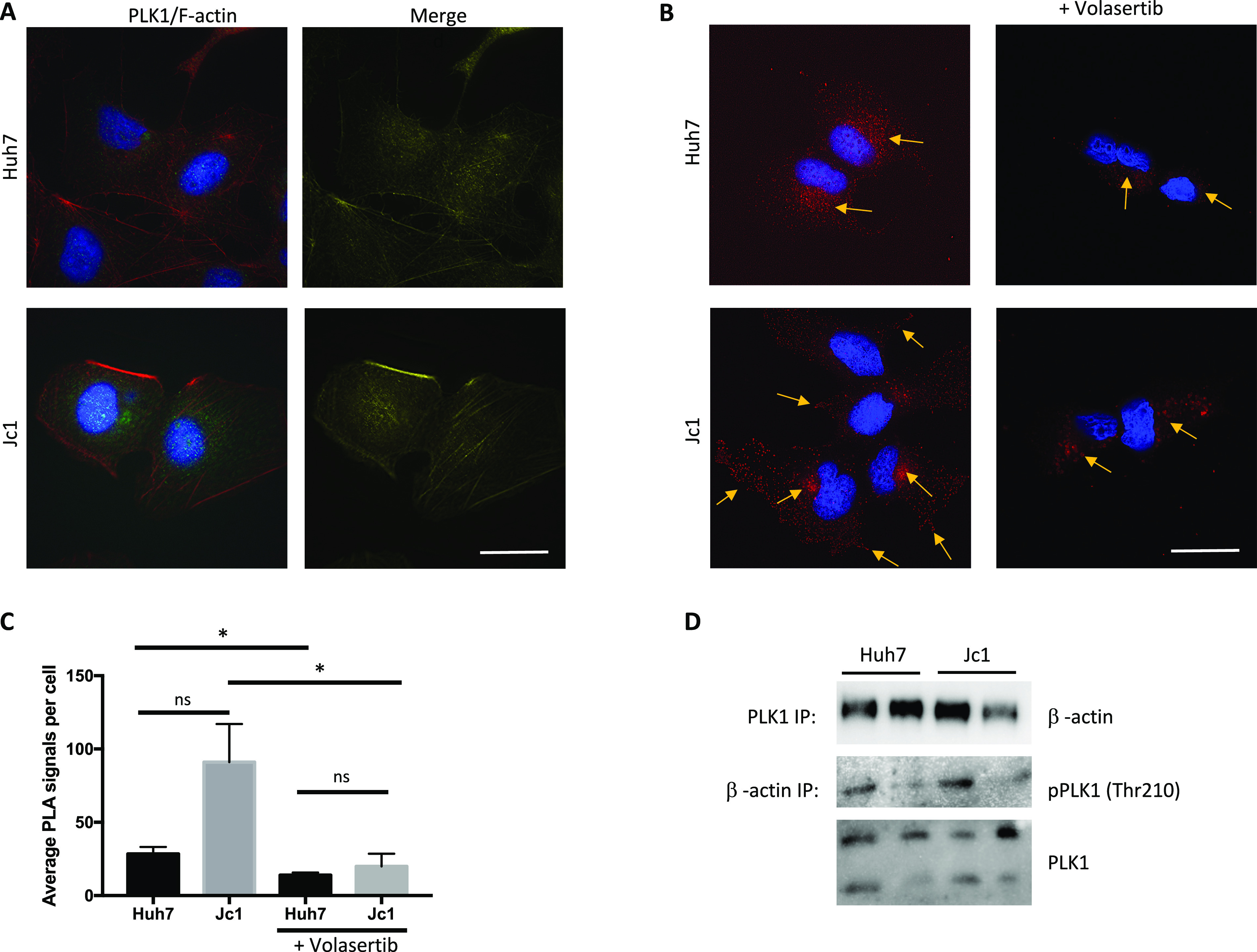
PLK1 interaction with the actin cytoskeleton in HCV-infected cells. **(A)** PLK1 (green) and β-actin (red) expression in HCV (Jc1)-infected cells with merged (yellow) PLK1 and β-actin channels. Fluorescent signals were deconvolved using the deconvolution analysis in the Softworx software. Representative images were shown. For each condition, more than 20 cells were observed. Scale bars represent 25 μm. **(B)** PLK1 and β-actin interaction in HCV (Jc1) infected cells by proximity ligation assay (PLA). PLA signal is detected as red spots of fluorescence, showing specific protein interaction in the cells (proteins are in close proximity, <40 nm). Yellow arrow denotes the localization of PLA spots on each respective cell. More than 10 random images were observed. Scale bars represent 25 μm. **(C)** Quantification of PLA red spots in each image using FIJI. Each bar represents the average of three experiments and error bars represent SEM. **(D)** Interaction of PLK1 and β-actin in HCV (Jc1)-infected cells as shown by coimmunoprecipitation. Representative images from two replicates are shown (*P < 0.05).

**Figure S5.**
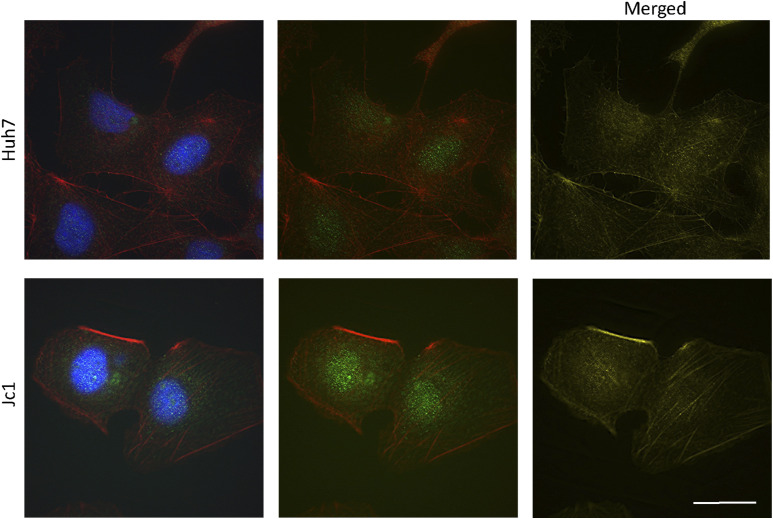
PLK1 expression in HCV-infected cells. Huh7 HCV-infected (Jc1) cells were labelled for PLK1 and F-actin. The left panels show PLK1 (green), F-actin (red), and nuclei (blue) channels. The middle panels show just PLK1 and F-actin channels. The right panels show merged (yellow) PLK1 and F-actin channels. Fluorescence signals were deconvolved using the Softworx software. Representative images are shown. For each condition, more than 20 cells were analysed. Scale bars represent 25 μm.

**Figure S6.**
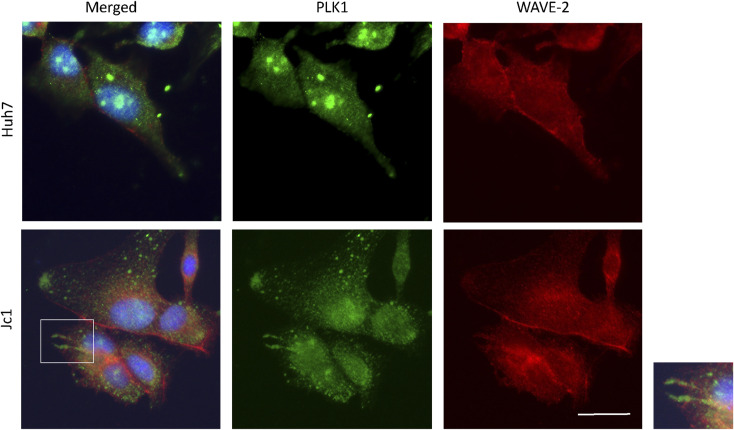
PLK1 and WAVE-2 expression in HCV (Jc1)-infected cells. Imaging results of PLK1 and WAVE-2 in HCV-infected cells. The left panel shows PLK1 (green), WAVE-2 (red), and nuclei (blue) channels, with specific channels for PLK1 (green) and WAVE-2 (red) in the middle panels. The right panel shows an enlarged view of the cell region inside the white box in the left panel, where PLK1 and WAVE-2 colocalized in the membrane protrusions of the cells. Representative images are shown. For each condition, more than 20 cells were analysed. Scale bar represents 25 μm.

Immunofluorescence microscopy demonstrated abundant PLK1 around the nucleus in both HCV-infected and-uninfected cells (Fig 5A). There appeared to be more colocalization between PLK1 and β-actin in infected cells, but it was not possible to quantify because of the diffuse signal. Separate PLK1 staining in HCV-infected cells showed that infected cells generally have more PLK1 around the nucleus and membrane protrusions of the cells (Figs S5 and S6).

Proximity ligation assay (PLA) was performed to quantify colocalization as it is more specific than confocal microscopy and detects when proteins are within 40 nm of each other (28). Colocalisation between PLK1 and β-actin was detected in the peri-nuclear region of uninfected cells, but in HCV (Jc1)-infected cells, it was seen throughout the cytoplasm, including the cell periphery and membrane protrusions (Fig 5B). This altered distribution was reversed by volasertib treatment. In HCV-infected cells, there was a trend towards an increased number of PLA spots (91.05 ± 25.99 versus 28.38 ± 4.81, *P* = ns; Fig 5C). Volasertib treatment reduced the average number of PLA spots in both infected and uninfected cells, to 19.97 (±8.54) and 14 (±1.67), respectively (*P* < 0.05).

Co-immunoprecipitation assays confirmed protein–protein interaction between PLK1 and β-actin in both infected and uninfected cells (Fig 5D). β-actin was detected in protein complexes pulled down by specific anti-PLK1 antibody, whereas PLK1 and phospho-PLK1 were detected in complexes precipitated by anti-β-actin antibody. Additional imaging of PLK1 and WAVE-2 (an actin polymerization factor which localizes to actin filaments) in HCV-infected cells showed that both PLK1 and WAVE-2 colocalized in membrane protrusions of the cells (Fig S6). Together, these results demonstrate that PLK1 interacts with β- actin filaments, allowing it to modify β-actin by phosphorylating S239.

### β-actin S239 phosphorylation alters actin dynamics and polymerization

**Figure S7.**
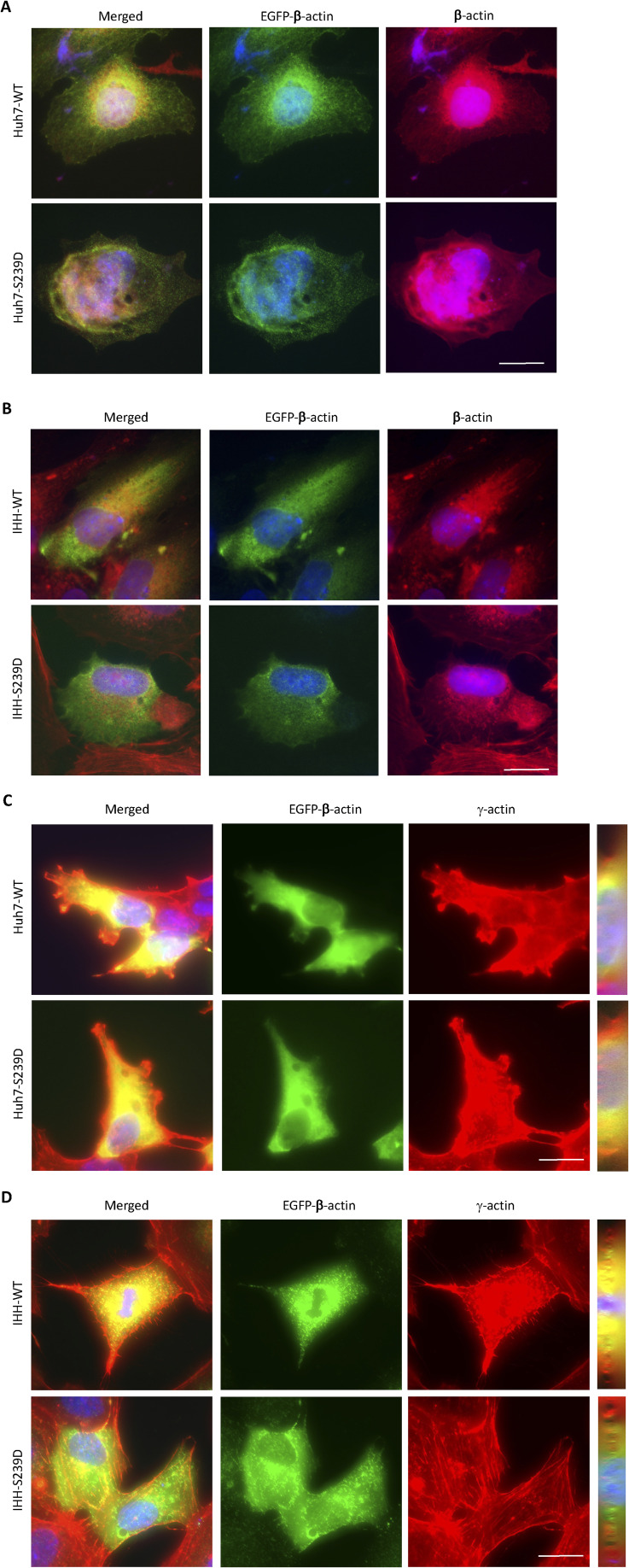
β- and γ-actin expression and localization in cells transfected with β-actin Ser239 phosphomimetic mutants. **(A, B)** β-actin expression and localization in Huh7s (A) and IHHs (B) transfected with WT or S239D β-actin–EGFP plasmids. Merged images show the expression of combined EGFP–β-actin and β-actin fluorescence. G-actin abundance can be seen around the nucleus, whereas F-actin is located more at the edge of the cells and in membrane protrusions. **(C, D)** γ-actin expression and localisation in Huh7s (C) and IHHs (D) transfected with WT or S239D β-actin–EGFP plasmids. The merged image is the maximum intensity projection image of all the Z slices. Merged images were z-sliced orthogonally to visualize specific labelling for γ-actin (red), β-actin (green), colocalisation (yellow), and nuclei (blue). Representative images of more than 20 cells from three independent experiments are shown. Scale bar represents 25 μm.

**Figure S8.**
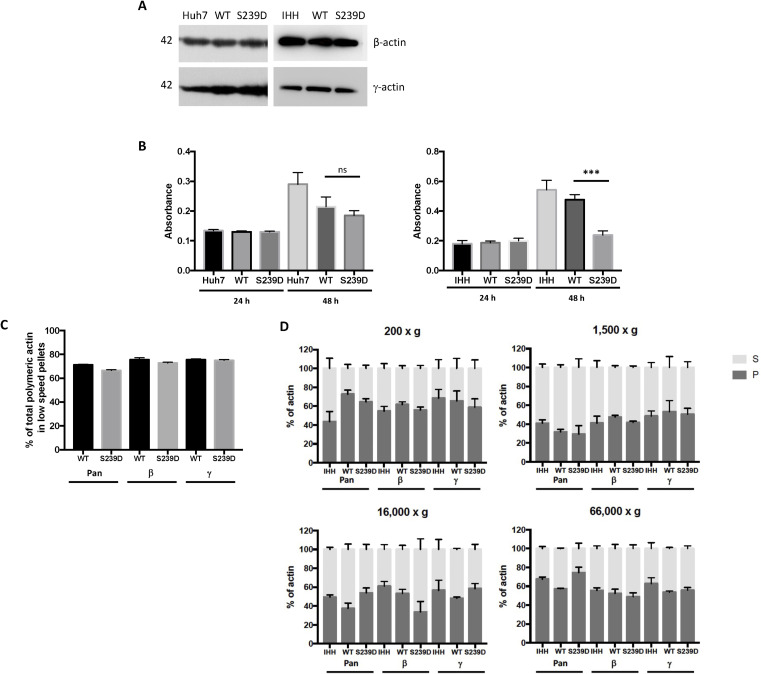
Actin protein expression, reduced cell proliferation, and actin biochemical fractionation from cells expressing a Ser239 phosphomimetic mutant form of β-actin. **(A)** Huh7 and IHH cells were transfected with WT or S239D β-actin–EGFP plasmids and harvested after 48 h. Lysates were analysed by Western blot and probed with specific antibodies for β-actin and γ-actin. A representative immunoblot from each group is shown, as the WT and S239D bands appeared similar, densitometry analysis was not performed. **(B)** Cell proliferation rate of Huh7 (left panel) and IHH (right panel) cells transfected with WT or S239D β-actin–EGFP plasmids after 24 and 48 h, as measured by Cell Proliferation BrdU ELISA kit. Absorbance value at 450 nm (OD450) correlates with cell proliferation rate. Error bar represents SEM. (*P < 0.05; **P < 0.01; ***P < 0.001; ns: not significant). **(C)** Percentages of total (pan), β- and γ-actin in the lower speed centrifugation fractions compared with the total amount of all pelleted actin fractions. **(D)** Biochemical fractionation of actin from lysates of non-transfected (IHH), WT–β-actin-EGFP (WT), and S239D–β-actin–EGFP mutant (S239D) transfected IHH cells. Proportion of actin (as a percentage) present in the pellet (F-actin, dark grey) and supernatant (G-actin, light grey) for β- and γ-actin isoforms and total (pan)-actin, estimated from quantification of Western blots using specific antibodies. Each bar represents average from two independent experiments.

Huh7 and immortalised human hepatocytes (IHH) cells expressing the activated (S239D mutant) form of β-actin showed perfect corelation of EGFP labelling with β-actin-specific staining, with no differences in β-actin distribution in transfected cells compared with non-transfected cells. However, not all γ-actin colocalised with GFP-tagged β-actin (Fig S7). No differences was observed between β-actin and γ-actin protein expression in nontransfected, WT-β-actin-EGFP (WT), and S239D-β-actin-EGFP mutant-transfected (S239D) Huh7 and IHH cells (Fig S8A). Cells expressing the activated (S239D mutant) form of β-actin have reduced cell proliferation after 48 h, which was more significantly observed in IHH cells (Fig S8B).

**Figure 6.**
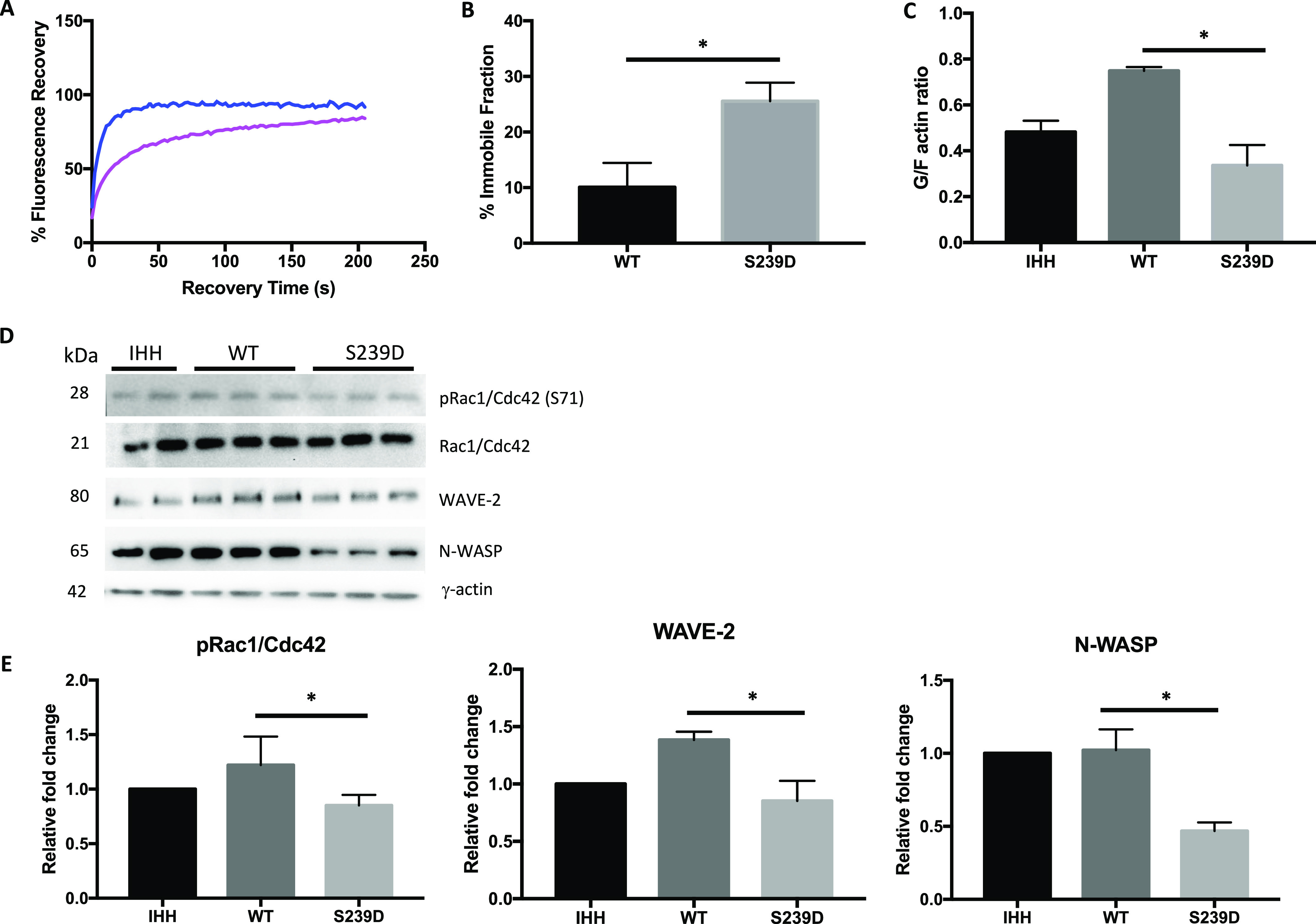
β-actin S239 phosphorylation effect on actin dynamics and polymerization. **(A)** FRAP analysis was performed on live immortalised human hepatocyte (IHH) cells expressing EGFP-tagged β-actin. The blue line is wild type (WT) β-actin, the pink line is the S239D mutant. The pre-bleach fluorescent ratio was set to 100% and the time for the first post-bleach image was set to 0 s. The recovery curves shown are an average of at least 10 cells from two experimental repeats. **(B)** The difference in percentage of immobile fraction between β-actin–S239D–EGFP and WT controls. Immobile fraction was determined by subtracting 1 from the mobile fraction (ratio between fluorescence intensity after full recovery and before photobleaching). **(C)** The ratio between the percentage of the supernatant containing G-actin and pellet containing F-actin from the 66,000*g* fraction. Actin fractionation was performed on lysates of non-transfected, WT–β-actin–EGFP (WT), and S239D–β-actin–EGFP mutant transfected (S239D) IHH cells. Actin proteins present in all the fractions were quantified by Western blots using antibodies to β-actin, γ-actin, and pan-actin. **(D)** Expression levels of pRac1/Cdc42 (Ser71), total Rac1/Cdc42, WAVE-2, and N-WASP in IHH cells transfected with WT or S239D β-actin–EGFP plasmids. A representative immunoblot from each respective group is shown. **(E)** Densitometry analyses of Western blots. Changes in protein expression are plotted relative to IHH non-transfected cells. For Rac1/Cdc42 expression, levels of expression were presented as ratio between phospho-Rac1/Cdc42 and total Rac1/Cdc42. Each bar represents the mean of three experiments and error bars represents SEM (*P < 0.05; ***P < 0.001).

**Figure S9.**
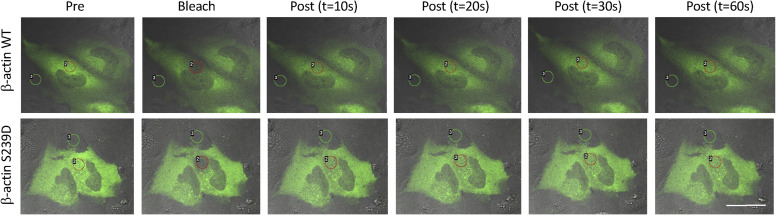
Time-course micrographs of IHH cells after photobleaching. Live-cell imaging of IHH cells transfected with WT–β-actin–EGFP or S239D–β-actin–EGFP mutant. Representative images are shown for pre-bleach, immediately after photobleaching, and 10, 20, 30, and 60 s after photobleaching. Red circles mark the photobleached regions in the perinuclear area and green circles are background. Scale bar represents 25 μm.

Confocal microscopy and FRAP analysis were performed on live cells expressing EGFP-tagged β-actin containing a phosphomimetic S239D mutation. To exclude possible effects of the EGFP tag on actin filament dynamics, a WT β-actin plasmid with EGFP tag was used as a control. Average transfection efficiency was ∼50–60%. IHH cells (a non-cancer cell line) were chosen for these experiments because of a more consistent cell morphology. There was reduced β-actin diffusion in cells expressing the S239D mutant (pink line) (r^2^ 0.781 versus 0.9229, *P* < 0.001; Fig 6A), with impaired fluorescence recovery (T_1/2_ 17.54 s versus 4.7 s) and lower final fluorescence intensity (FI) (79.2% versus 92.9%). Time-course micrographs for both WT and S239D cells are shown in Fig S9.

The immobile or retained β-actin pool is calculated by measuring the difference between the final plateau of FRAP recovery curves and the pre-bleach fluorescence values. In cells expressing the S239 mutant, the fluorescence recovery curve reached only ∼70% of the pre-bleach ratio, compared with ∼90% for WT control cells, indicating a higher immobile β-actin fraction in S239 cells (25.53% ± 3.36 versus 10.09% ± 4.35, *P* = 0.04; Fig 6B).

The slower fluorescence recovery in S239D cells suggests that more of the actin is present in stable filament (F)-actin complexes, with less globular (G)-actin available for diffusion or exchange, giving a lower G/F actin ratio. To test this hypothesis, the relative compositions of G- and F-actin were measured, with or without the phosphomimetic S239D mutation. Biochemical fractionation by serial centrifugation of lysates demonstrated a lower actin G/F ratio in S239D cells compared with WT controls (0.33 ± 0.08 versus 0.75 ± 0.02, *P* < 0.05; Fig 6C). Consistent with this, after fractionation at the highest sedimentation speed (66,000*g*), S239D cells retained 74.4% of total actin in the pellet, compared with 57.2% for WT controls (Table S1 and Fig S8C and D).

Table S1. Proportion of actin isoforms in IHH lysates after biochemical fractionation. Proportion of actin (as a percentage) in the supernatant and pellet, at different centrifugal steps, during fractionation of cell lysates from non-transfected (IHH), WT–β-actin–EGFP (WT), and S239D–β-actin–EGFP mutant (S239D)-transfected IHH cells. Pellet represents F-actin and supernatant represents G-actin monomer.

**Figure S10.**
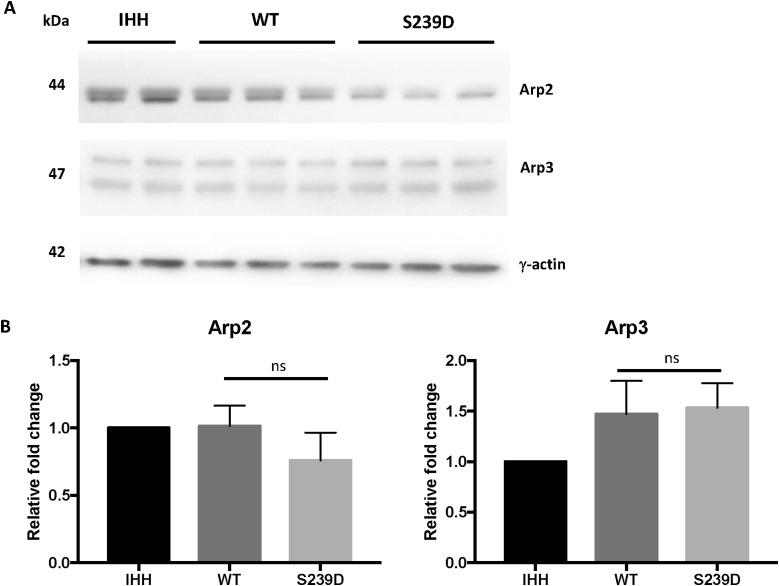
Expression of actin polymerization factors in IHH cells expressing a Ser239 phosphomimetic mutant form of β-actin. **(A)** IHH cells were transfected with WT or S239D β-actin–EGFP plasmids and harvested after 48 h. Lysates were analysed by Western blot and probed with specific antibodies for Arp2 and Arp3. γ-actin was used as the loading control. A representative immunoblot from each group is shown. **(B)** Densitometry analyses of Western blots. Changes in protein expression are plotted relative to IHH non-transfected cells. Each bar represents the mean of three experiments. Error bar represents SEM (*P < 0.05; **P < 0.01; ***P < 0.001; ns, not significant).

Expression of actin polymerization factors was measured in cells expressing the activated (S239D) form of β-actin or WT β-actin, and in non-transfected controls (IHH). There was no difference in the total expression of the small GTPase actin regulator Rac1/Cdc42 (Fig 6D), but activation of Rac1/Cdc42 was reduced in S239D cells, with a reduced ratio of phosphorylated Rac1/Cdc42 (Ser71) to total Rac1/Cdc42 (0.67 ± 0.05 versus 0.92 ± 0.04, *P* < 0.05; Fig 6E). Among downstream effectors of Rac1/Cdc42, the actin nucleation-promoting factors WAVE-2 (0.85 ± 0.17 versus 1.38 ± 0.07, *P* < 0.05) and N-WASP (0.47 ± 0.06 versus 1.02 ± 0.14, *P* < 0.05) were significantly reduced in S239D cells compared with WT controls (Fig 6D and E). In S239D cells, there was also a trend towards reduced levels of actin nucleation and polymerization factors Arp2 and Arp3, both downstream from WAVE-2/N-WASP (Fig S10).

In sum, reduced Rac1/Cdc42 activation and expression of actin polymerization initiation factors (WAVE-2 and N-WASP) in cells expressing S239D-β-actin suggest a reduced rate of actin polymerization.

### β-actin S239 phosphorylation induces changes in cell morphology

**Figure 7.**
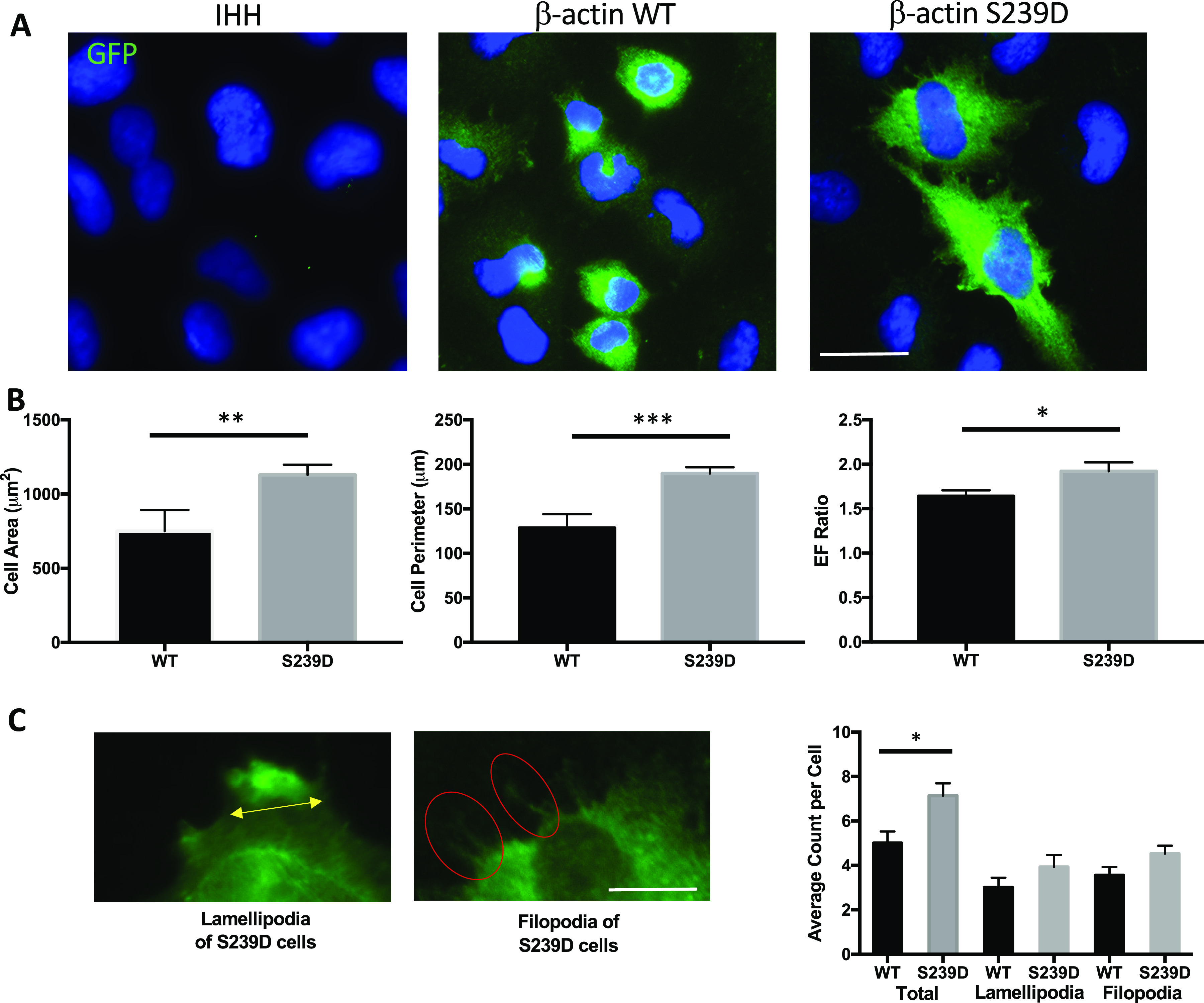
Morphological changes in cells expressing a β-actin S239D phosphomimetic mutant. **(A)** IHH cells transfected with WT–β-actin–expression of green fluorescence protein (EGFP) or S239D–β-actin–EGFP plasmids, showing EGFP which is not present in non-transfected cells. Scale bar represents 25 μm. **(B)** Measurement of cell area, cell perimeter, and EF ratio in S239D versus WT IHHs using FIJI. Each bar represents an average of at least 30 cells; values from three independent experiments were recorded and averaged. **(C)** Membrane protrusions of β-actin–EGFP IHHs. Yellow line denotes lamellipodia and red circle encircles filopodia. Average number of membrane protrusions per cell was counted in total, and for lamellipodia and filopodia, separately. Scale bar represents 100 μm. Error bar represents SEM (*P < 0.05; **P < 0.01; ***P < 0.001).

The S239D cells had altered cell morphology compared with WT controls (Fig 7A), with larger average cell area (1,129.5 ± 108.1 versus 750.8 ± 197, *P* < 0.01) and increased cell perimeter (189.7 ± 11.7 versus 128.4 ± 21.7, *P* < 0.001; Fig 7B). The elliptical factor (EF) ratio between the longest and shortest axes of the cells was also increased by ∼17% in S239D cells (1.92 ± SEM 0.1 versus 1.64 ± SEM 0.07, *P* < 0.05; Fig 7B), suggesting a more polarized phenotype. S239D cells had a higher average number of total membrane protrusions (7 ± 0.56 versus 5 ± 0.53, *P* < 0.05; Fig 7C), with a trend towards more lamellipodia (Fig 7C, 4 ± 0.54 versus 3 ± 0.45; *P* = ns) and filopodia (4.5 ± 0.35 versus 3.5 ± 0.38; *P* = ns).

### β-actin S239 phosphorylation increases cell migration

**Figure 8.**
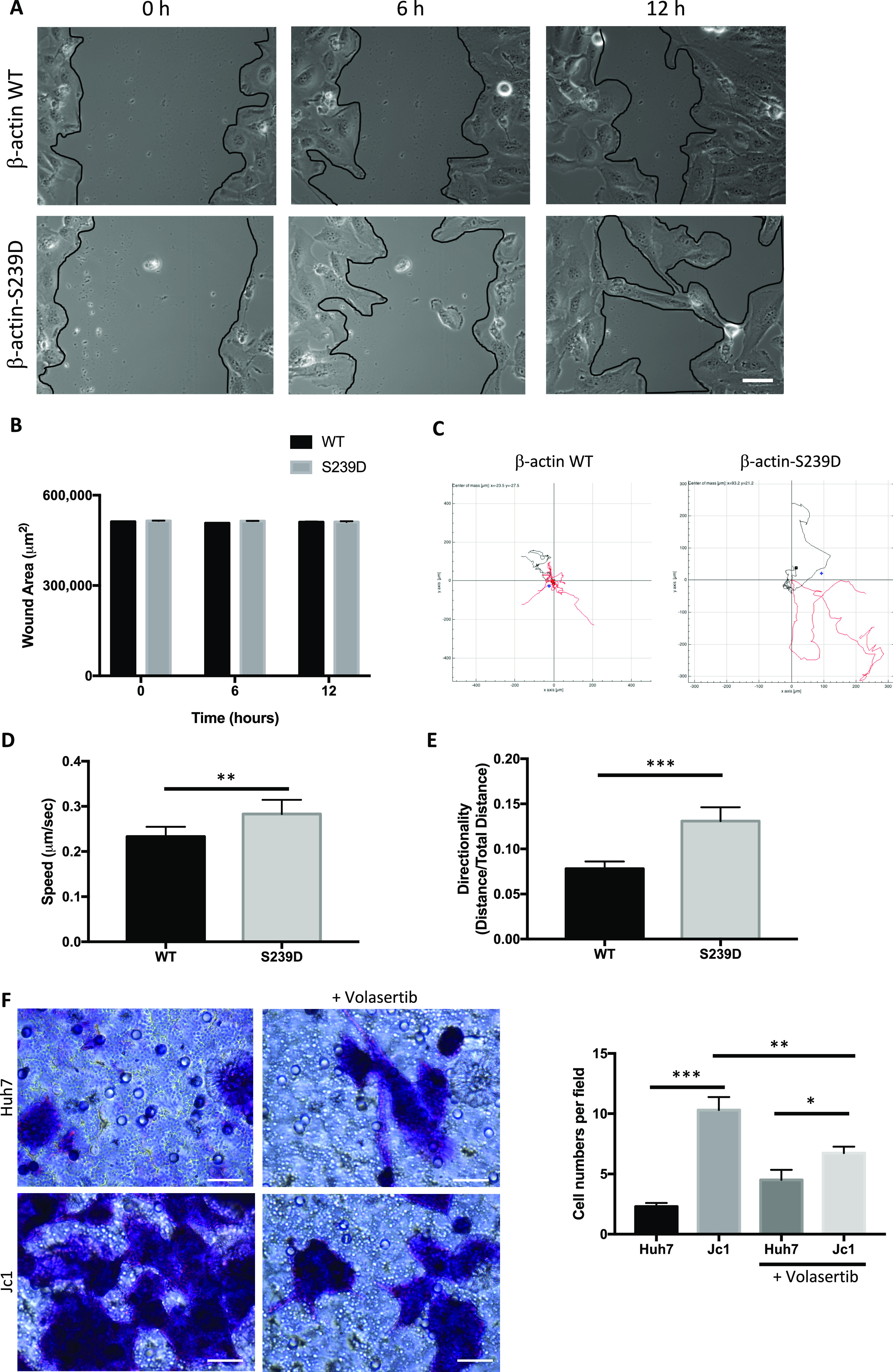
Cell migration is regulated by PLK1-mediated phosphorylation of β-actin at Ser239. **(A)** IHH cells were transfected with either WT-β-actin-EGFP or S239D-β-actin-EGFP plasmids. Cell monolayers were scratched with a sterile pipette tip to generate a wound. Representative images at 0, 6, and 12 h are shown from three independent experiments. Scale bars represent 50 μm. **(B)** Changes in the wound area were measured at each time point. Each bar represents the average of three independent experiments. Error bars represents SEM. **(C)** Migrating cells from each wound edge were tracked at 5-min interval for 12 h. Representative examples of individual migration tracks of IHH-WT and S239D cells. **(D)** Quantitation of migration speed of IHH-WT and S239D cells. **(E)** Quantification of directionality of cell migration was calculated as the linear distance (D) over the total track distance (T) of a cell. Values from at least 30 cells from three independent experiments were recorded and averaged. Error bars represents SEM. *P*-values less than 0.05 indicate statistical significance. **(F)** Uninfected and HCV (Jc1)-infected cells resuspended in serum-free media were seeded onto the membrane of the Transwell insert. After 24 h, complete media, with or without volasertib IC50 concentration (6.28 μM), were added to the lower chamber. After another 24 h, migrating cells were stained with crystal violet and counted. Volasertib treatment reduced cell migration in HCV (Jc1)-infected cells. Scale bars represent 100 μm. Graph on the right panel shows the average number of migrated cells in each insert after 24 h (*P < 0.05; **P < 0.01; ***P < 0.001).

To examine the effect of β-actin phosphorylation on cell migration, a cell wound closure assay was used. IHHs were seeded onto four-well chambered slides and after 24 h transfected with β-actin-EGFP plasmids (S239D phosphomimetic mutant or WT control). 24 h after transfection, a wound was created by scratching the monolayer, then cells surrounding the wound were imaged every 5 min up to 12 h, with representative images shown at 0, 6, and 12 h (Fig 8A). No difference in wound closure rate was observed in cells transfected with S239D or WT β-actin–EGFP plasmids (Fig 8B).

Time-lapse imaging was performed over 12 h, individual cells were tracked using the FIJI Manual Tracking plugin, and migration tracks were plotted using the Chemotaxis Tool plugin. Both S239D and WT cells moved perpendicular to the edges of the wound into the open area, but S239D cells tended to change direction more often and moved greater distances (Fig 8C). S239D-expressing cells had a higher average speed (0.39 ± 0.04 versus 0.25 ± 0.02, *P* < 0.01; Fig 8D) and higher calculated directionality, which is the linear distance (D) divided by the total track distance (T) (0.15 ± 0.02 versus 0.06 ± 0.01, *P* < 0.001; Fig 8E).

### Increased cell migration in HCV-infected cells is reversed by the PLK1 inhibitor volasertib

HCV (Jc1)-infected Huh7 cells and uninfected controls were seeded into Transwell inserts in serum-free media and the lower well was filled with complete media, either with or without volasertib (6.28 μM). After 24 h, cells were fixed with crystal violet and counted by microscopy (Fig 8F). HCV-infected cells had a higher average number of migrating cells than controls, both in complete (10.3 ± 1.08 versus 2.3 ± 0.3, *P* < 0.001) and in volasertib-containing media (6.73 ± 0.54 versus 4.5 ± 0.85, *P* < 0.05). Volasertib treatment reduced the migration of HCV-infected cells (*P* < 0.001; Fig 8F), but not uninfected controls, excluding a reduction simply because of volasertib toxicity. These observations confirm that HCV-infected cells have increased migratory ability, which is significantly reduced after inhibition of PLK1 by volasertib.

## Discussion

HCC associated with viral hepatitis is a leading cause of cancer-related mortality, but the mechanisms of cancer development are not clear (29). In addition to nonspecific factors such as inflammation and cirrhosis, virus-specific factors play a role as HCC risk in people with HCV-induced cirrhosis is twofold to threefold higher than with cirrhosis because of alcohol or MAFLD (10). This suggests that virus-induced premalignant changes confer a survival advantage for infected cells that are amplified in the presence of liver inflammation and hepatocyte turnover, as confirmed for HBV (30). The resulting clonal expansion of premalignant cells giving rise to monoclonal liver nodules that persist after HCV cure confers an ongoing risk of HCC (9).

We identify a novel mechanism for HCV-induced promotion of HCC through posttranslational modification of the cytoskeletal protein β-actin. β-actin plays a key role in maintaining cell shape and mediates cell motility through dynamic polymerization/depolymerization of the actin cytoskeleton (31, 32). This process is regulated by Rho GTPases and other actin-binding proteins (33). We show that actin dynamics is regulated by direct phosphorylation of β-actin at Ser239 in the context of HCV infection. The infected cells have an increased abundance of F-actin filaments and reduced G-actin suggesting Ser239 phosphorylation induces actin polymerization. We propose that this phosphorylation of β-actin stabilises actin filaments, reducing the need for de novo actin polymerization through the Rac1–WAVE–Arp2/3 complex cascade.

Several viruses including HCV have been reported to subvert the actin cytoskeleton to support their own replication (34, 35, 36). HCV nonstructural proteins NS3 and NS5A interact with actin polymers to facilitate transfer of the viral replication complex to different parts of the cell (36, 37). Similarly, other viruses including HBV can activate the Rho-family GTPase Rac1 to promote viral replication (37). However, identification of Ser239 phosphorylation of β-actin in HCV-infected cells is novel and this is the first report of a direct virus-induced modification.

A key step in cancer progression is increased cell motility enabling metastasis and tumour invasion. PLK1 is a major regulator of mitosis and cytoskeletal dynamics and is associated with poor prognosis and metastasis in a range of cancers including HCC, non-small cell lung cancer, breast, ovarian, squamous cell carcinomas, melanoma, and large B cell lymphoma (12, 13). PLK1 is a known key regulator of cytoskeletal dynamics during HCC progression (15) and is overexpressed in patients with HCC who have vascular invasion (16).

We identified PLK1 as the kinase mediating β-actin phosphorylation at S239 using both in silico and in vitro approaches. In the context of HCV infection, PLK1 supports production and secretion of virus particles by interacting with and hyper-phosphorylating the key viral protein NS5A (38, 39). In turn, NS5A stimulates PLK1-mediated phosphorylation of several cellular proteins (18), and here, we show that PLK1 mediates phosphorylation of β-actin. We observed the direct interaction between PLK1 and β-actin and localization of PLK1 to actin protrusions, neither of which has been described before (12, 40).

Our findings implicate PLK1-induced β-actin remodelling as an important mechanism in HCV-induced liver cancer progression. Consistent with this and with data from GEO datasets, in functional experiments we showed that phosphorylation of Ser239 influences β-actin polymerization and distribution, resulting in increased cell motility and directional migration. Importantly, these changes were prevented when cells were treated with the PLK1 inhibitor volasertib.

Recent studies suggest that virus-induced HCC (because of HBV or HCV) behaves differently to tumours associated with MAFLD or alcohol (41). Consistent with this, our data suggest that PLK1 may also contribute to HCC progression in patients with hepatitis B as we showed increased PLK1 expression in their livers. HBV activates PLK1 in vitro and in mouse models promoting formation of HBV capsids (42). The HBV X protein can promote HCC by activating PLK1, attenuating the DNA damage checkpoint and inhibiting DNA repair (43). This oncogenic transformation can be reduced by inhibiting PLK1 (44).

Our findings have important clinical ramifications as they provide a rationale for targeting PLK1 to treat HCC and support early treatment to prevent tumour progression, particularly in patients with virus-induced HCC. Because PLK1 regulates cell motility, it suggests that in addition to inhibiting cell proliferation, targeting PLK1 reduces tumour-cell migration. We propose that PLK1 inhibitors should be given early in patients with HCC to reduce tumour invasion and metastasis. Several PLK1 inhibitors including volasertib (BI6267) have shown encouraging results for a range of tumours (12), including in vitro efficacy against HCC (45). In a recent Phase 1 clinical trial for patients with metastatic HCC, siRNA against *PLK1* was given as a lipid nanoparticle formulation (TKM-080301) and was well tolerated (46). PLK1 inhibitors have a favourable safety and tolerability profile making them ideal for combination treatment with traditional chemotherapy agents, or as long-term treatment to prevent tumour progression (47).

In conclusion, we provide new insights into HCC pathogenesis and the regulation of tumour cell motility, particularly in the context of viral hepatitis. Our findings suggest that early treatment with PLK1 inhibitors in combination with other agents may reduce HCC progression, invasion, and metastasis.

## Materials and Methods

### Cell lines and plasmids

Cell lines used were human Huh7 hepatoma cells and IHHs. Both cell lines were incubated in normal culture conditions (37°C, 5% CO_2_). A plasmid encoding the HCV Jc1 genome was a gift from Dr. Thomas Pietschmann (TWINCORE) (48). Plasmids encoding HCV JFH1/GND (a replication incompetent mutant) were a kind gift from Prof Takaji Wakita (National Institute of Infectious Disease, Tokyo, Japan) (49). pEGFPN1 was purchased from OriGene. HCV infection was established by electroporating viral RNA transcribed from HCV plasmids (49). Infection was confirmed 48 h after electroporation by immunolabelling for HCV NS5A.

### Volasertib treatment

The volasertib concentration required to inhibit growth by 50% (IC50) was determined using crystal violet assay. Huh7 cells were treated with increasing doses of volasertib, ranging from 100 nM to 50 μM for 24 h, with DMSO as positive control. The viable cell percentage on treated Huh7s was determined by comparing the average OD488 values of treated with non-treated cells. The percentage cell death was calculated by subtracting the viable cell percentage from 100% (50). The IC50 value was calculated from the survival curves using the Bliss method (51).

### Antibodies

Antibodies were used for both Western blots and immunofluorescence. Primary antibodies for Arp2, Arp3, α-tubulin, β-actin, E-cadherin, KinomeView Profiling kit, N-WASP, pan-actin, phospho-PLK1 (Thr210), PLK1, phospho-Rac1/Cdc42(Ser71), Rac1/Cdc42, vimentin, and WAVE-2 were purchased from Cell Signaling Technology (#3128, #4738, #2144, #4970, #3195, #9812, #4848, #8456, #9062, #4513, #2461, #4651, #5741, and #3659). PLK1 primary antibody used for immunofluorescence was purchased from Thermo Fisher Scientific (#37-7100); γ-actin antibody was purchased from Abcam (#ab123034). HCV NS5A sheep polyclonal antibody and NS5A mouse antibody were gifts from Professor Mark Harris (University of Leeds, UK) and Associate Professor Michael Beard (University of Adelaide), respectively. Secondary antibodies were either conjugated with HRP; anti-rabbit Ig/HRP (#P0448), and anti-mouse Ig/HRP (#P0161) (DAKO) or labelled with fluorescence dyes: antirabbit IgG Alexa Fluor 488 (#A21206), anti-mouse IgG Alexa Fluor 488 (#A21202), anti-mouse IgG Alexa Fluor 594 (#A21203), and anti-rat IgG Alexa Fluor 647 (#A21472) (Thermo Fisher Scientific).

### RNA extraction and quantitative real-time PCR

Total cell RNA was isolated using FavorPrep Tissue Total RNA Mini Kit (Favorgen) according to the manufacturer’s instructions. Eluted RNA was quantified using a NanoDrop ND100 spectrophotometer and stored at −80°C. cDNA was reverse transcribed from total RNA using MMLV reverse transcriptase (Promega) according to the manufacturer’s instructions. Quantitative real-time PCR was performed using SYBR Green PCR Master Mix (Thermo Fisher Scientific) on a Rotor-Gene 6000 (Corbett Research). In brief, the reaction mixture (20 μl total volume) contained 500 ng cDNA, 0.2 μM forward and reverse primers, and 5.5 μl SYBR Green PCR Master Mix. Thermal cycling conditions were 95°C for 10 s followed by 40 cycles at 95°C for 5 s and 60°C for 30 s. Experiments were performed in triplicate.

### Protein extraction and Western blot analysis

Cellular protein was extracted from cell monolayers using RIPA (radioimmunoprecipitation assay) buffer supplemented with complete protease inhibitor cocktail (Roche), phosphatase inhibitor cocktail 2 (Sigma-Aldrich), PMSF, and sodium fluoride (NaF). Cell pellets were resuspended in RIPA buffer, incubated on ice for 30 min, and centrifuged at 20,000*g* at 4°C for 10 min. Protein concentration was determined using the DC protein assay (Bio-Rad) with BSA as a standard. SDS–PAGE gels were prepared by casting 10–12% separating gels and 5% stacking gels. Protein samples (30 μg in 1x Laemmli buffer) were denatured in 95°C for 5 min and separated by electrophoresis at 100 V for 1 h. The separated proteins were transferred onto PVDF membranes in Tris-glycine and methanol transfer buffers. The membranes were blocked overnight at 4°C in 5% skim milk in TBS and then incubated with 1:1,000 dilution of primary antibodies in a blocking buffer, overnight at 4°C. The membranes were washed in TBS and 0.05% Tween-20 and incubated with 1: 10,000 dilution of HRP-conjugated secondary antibodies in the blocking buffer for 1 h at room temperature. Protein bands were detected using 1:10 mixture of SuperSignal West Femto Maximum Sensitivity Substrate and SuperSignal West Pico Chemiluminescent Substrate (Thermo Fisher Scientific) on ChemiDoc Touch Imaging System (Bio-Rad). Band intensity was quantified using FIJI (ImageJ) software (52).

### 2-D gel electrophoresis

Cells were washed three times in ice-cold PBS, scraped off the flasks, and pelleted by centrifugation at 3,000*g* for 10 min. Protein yield was estimated using the 2D Quant Kit (GE Healthcare). 100 μg of protein from the cell pellet was dissolved in 1:1 mixture of the extraction buffer (8 M urea, 2 M thiourea, 25 mM Tris-base, and 4% [wt/vol] CHAPS) and the extraction buffer with 1% carrier ampholytes (3/10; Bio-Lyte). Samples were further treated with 2.3 mM TBP/45 mM DTT for 1 h at 25°C and 230 mM acrylamide for 1 h at 25°C. Treated protein samples (125 μl) were loaded into lanes of an Immobiline DryStrip rehydration tray to rehydrate the immobilized pH gradient (IPG) 7 cm (pH 3–10) strip for 16 h. IPG strips were transferred to an Ettan IPGPhor II Isoelectric Focusing System (GE Healthcare) for first-dimension isoelectric focusing up to 37,500 Vh at 17°C. Separation in the second dimension was performed using 12.5% separating gel and 5% stacking gel at 90 V, 4°C, overnight. Gels were fixed the next day with 10% methanol and 7% acetic acid for 1 h with continuous shaking. Gels were rinsed thrice with distilled water for 30 min before stained with Coomassie brilliant blue colloidal solution (2% phosphoric acid, 10% ammonium sulphate, 0.1% Coomassie brilliant blue, and 20% methanol in water) for 16–20 h with continuous shaking. Gels were washed with 0.5 M NaOH thrice for 15 min and twice for 30 min with continuous shaking. Protein patterns in the gels were recorded as digitised images using a Typhoon Trio scanner (GE Healthcare).

### MALDI TOF-MS protein identification

2-D gel electrophoresis spots of interest were excised and transferred into 1.5 ml sterile LoBind tubes (Eppendorf) for in-gel protein digestion. Gel pieces were de-stained with 100 μl of 50% (vol/vol) ACN/50 mM ammonium bicarbonate (NH4HCO3) for 10 min at room temperature on tube-rotating mixers. De-staining was repeated for at least three times or until the gel pieces became colourless. Gel pieces were dehydrated in 100% acetonitrile and dried in a Concentrator plus vacuum centrifuge (Eppendorf). The dried gel pieces were rehydrated with 50 μl of a 1:40 dilution of trypsin (15 ng/μl, sequencing grade) in 50 mM NH4HCO3 and incubated at 4°C for 1 h. After digestion, excess trypsin was removed and 20 μl of 50 mM NH4HCO3 was added to cover the gel pieces. The tubes were then incubated at 37°C for 16 h. Final protein extraction was performed on ice by sonication as follows: 2 s (sonication) and 4 s (rest) for 20 min in total. The supernatant was then dried using a Concentrator plus vacuum centrifuge (Eppendorf) at 45°C for 20 min. MALDI TOF (matrix-assisted laser desorption ionization–time of flight) mass spectrometry was performed using a TOF/TOF 5800 system (AB SCIEX) for peptide mass fingerprinting and protein identification.

### Construction and transfection of β-actin–EGFP plasmids

The human β-actin coding region (1,128 bp) was amplified from DNA extracted from Huh7 cells using the β-actin cloning (ACTB-CL) primer sets with 1 U/μl KOD hot start DNA polymerase (Novagen). A PCR-based, site-directed mutagenesis strategy was employed to create a point mutation at β-actin codon 239 (AGC to GAC), changing the amino acid from serine (S) to aspartic acid (D). Two β-actin fragments were synthesized in separate reactions by combining β-actin mutation (ACTB-MT) primers with the primers for the full-length cDNA using Blend Taq PCR and combined into a full-length mutant fragment with LA Taq PCR. β-actin and S239D-β-actin PCR products and pEGFP-N1 were purified and double-digested with *Bam*HI and *Xho*I (Promega) then ligated using T4 DNA ligase (NEB). The ligated product was cloned into XL10-Gold Ultracompetent Cells (Stratagene). Transformed cells were selected on LB agar with kanamycin (50 μg/ml) at 37°C. Colonies with suitable plasmids were selected and used for DNA plasmid extraction using Plasmid Maxi Kit (QIAGEN). The β-actin sequence in the final-expression plasmids was verified by DNA sequencing. β-actin–EGFP plasmids were transfected into Huh7s and IHHs using FuGENE HD Transfection Reagent (Promega) (using 4.5:1 ratio in Opti-MEM), according to the manufacturer’s protocol.

### Co-immunoprecipitation

Cell lysates (200 μl) were mixed with 1 μl primary antibody and incubated overnight at 4°C with gentle rocking. Proteins were co-precipitated with 20 μl of recombinant protein g-sepharose 4B conjugate for 3 h with gentle rocking at 4°C. Lysates were pelleted by 15,000*g* for 30 s at 4°C and washed five times with 500 μl 1% Triton X-100 in PBS. Lysates were kept on ice during washes. Samples were centrifuged 15,000*g* for 5 min and supernatants were discarded. Pellets were suspended in 1x Laemmli buffer, denatured at 95°C for 5 min, and lysates were analysed by Western blotting.

### Cell proliferation assay

Cell proliferation assays were performed using a Cell Proliferation BrdU ELISA kit (Roche), which measures incorporation of BrdU during DNA synthesis. In brief, cells were cultured in 96-well microplates in a final volume of 100 μl/well. BrdU was added to the cells and the cells incubated for 2–24 h. Media were removed, and cells fixed with Fix/Denat solution for 30 min. Anti-BrdU-POD was then added to the cells and incubated for 90 min. After washing three times with a washing solution, the cells were incubated with a substrate solution for 30 min. The immune complex-substrate reaction was stopped by adding 1 M H2SO4 for 1 min with gentle agitation. The reaction product was quantified by measuring absorbance at 450 nm using a Victor^3^ Multilabel Plate Reader (Perkin Elmer).

### Transwell cell migration assay

Confluent cells were trypsinised and pelleted. Cells were then resuspended in serum-free media containing 0.1% BSA. One hundred microlitres of cell suspension (0.1 x 10^6^ cells/ml) was added to the top of the filter membrane (8.0 μm pore size) in a Transwell insert and incubated for 10 min at 37°C. Next, 600 μl of complete media, either with or without volasertib (IC50 value of 6.28 μM, based on crystal violet IC50 assay) was added carefully into the bottom of the lower chamber, in 24-well plates. The Transwell inserts were removed from the plate after 24 h and fixed with 0.2% crystal violet/methanol mix for 10 min at room temperature. Excess crystal violet was removed, and the inserts were washed with distilled water and then air-dried. The number of migrated cells was then counted for each well.

### Wound closure assay

IHH were seeded onto four-well chambered slides (cat #155383; Thermo Fisher Scientific] at a density of 0.05 x 10^6^ cells/well and transfected with β-actin–EGFP plasmids the next day. The confluent monolayer 24 h post-transfection was disrupted with a cell scraper. Images of the wound area were acquired every 5 min up to 12 h using a Zeiss AxioVert 200 M Live Cell Imaging System (Zeiss), with 10x A-Plan 0.25 Ph1 objective. The rate of wound closure was calculated by determining the average wound area at every time point. Experiments were performed in triplicate, and three random fields from each well were recorded. The time-lapse videos were used for further analysis using FIJI plugins. The Manual Tracking plugin was used to follow migration tracks of individual cells from the first slice (0 h) to 144^th^ slice (12 h) and determine average velocity and directionality. The Chemotaxis Tool plugin was used to plot the migration tracks and to see changes in direction over time.

### FRAP assay

Cells were seeded onto four-well Nunc chambered slides (cat#155383; Thermo Fisher Scientific) at a density of 0.05 x 10^6^ cells/well and transfected with β-actin–EGFP plasmids. 2 d after transfection, FRAP analysis was performed using a FV 1000 Confocal Laser Scanning Microscope (Olympus) with 60x water objective. FRAP analysis was performed by taking five control images before bleaching, bleaching the cell of interest at 100% laser transmission (488 nm), and capturing a series of images immediately after bleaching. Images were captured every 1 s for 100 s after bleaching. FRAP rate was calculated as described previously (53, 54). Fluorescence data before and after bleaching were expressed as a ratio to normalise and compare data between cell samples. The pre-bleach ratio was set to 100% and the time for the first post-bleach image was set to 0 s. The average data for at least 10 cells from two experimental repeats were plotted. The fluorescence recovery curve was plotted in Graph Pad Prism 7.0 using single association curve, with the curve weighted by 1/Y2. The mobile fraction (fm) is calculated by dividing the fluorescence intensity after full recovery (F_∞_) to fluorescence intensity before photobleaching (F0) which were set to 100%. The immobile fraction (fi) was obtained by subtracting 1 with fm value.

### Immunofluorescence

Cells were cultured on top of sterilized coverslips fitted on a 24-well plate and fixed with either 4% PFA for 10 min at room temperature or methanol for 20 min at −20°C. Cells were permeabilised and blocked using 0.1% Triton X-100 for 10 min and 5% FBS/PBS for 1 h at room temperature. Primary antibody incubations were performed in the blocking solution for 1 h at room temperature or overnight at 4°C, whereas secondary antibody incubations were performed in the blocking solution for 1 h in the dark. Actin filaments were labelled with β-actin-specific antibody for 40 min or phalloidin stain for 20 min. Coverslips were mounted with ProLong Gold antifade reagent with DAPI (4’,6-diamidino-2-phenylindole) (Thermo Fisher Scientific). Immunofluorescence images were collected using a 60x/1.42 Oil Plan APO objective on a DeltaVision Elite High-Resolution Microscope (GE Healthcare) and softWoRx software (Applied Precision). Images were analysed using FIJI software. The average number of membrane protrusions was counted for each cell after image thresholding adjustment using FIJI. Actin protrusions were identified manually using standard definitions: lamellipodia are membrane protrusions ≥5 μm in width, whereas filopodia are finger-like protrusions with width ≤4 μm (33).

### PLA

Duolink PLA (Sigma-Aldrich) was performed according to the manufacturer’s instruction, to detect, quantify, and determine cell localisation of protein–protein interactions. Immunofluorescence images were collected using a 60x/1.42 Oil Plan APO objective on a DeltaVision Elite High-Resolution Microscope (GE Healthcare) and softWoRx software (Applied Precision). PLA spots were counted using the Find Maxima options in FIJI.

### Biochemical fractionation of actin and estimation of globular- and filamentous-actin ratios

Biochemical fractionation of actin was performed as described previously (55). Cells were harvested and lysed in filamentous (F)-actin stabilization buffer (50 mM PIPES pH 6.9, 50 mM NaCl, 5 mM MgCl2, 5 mM EGTA, 5% glycerol, 0.1% NP-40, 0.1% Triton X-100, 0.1% Tween 20, 0.1% 2-mercaptoethanol, 1 mM ATP, and PMSF). Lysates were sedimented at 37°C by serial centrifugation at 200*g* for 5 min, 1,500*g* for 15 min, 16,000*g* for 15 min, and 66,000*g* for 1 h. Protein concentration in all fractions were normalized by estimated protein concentration in the 200*g* supernatant. Aliquots of supernatant and pellet fractions from each centrifugation steps were analysed by Western blot. Actin proteins present in all the fractions were quantified by Western blots using specific antibodies against βactin, γ-actin, and pan-actin.

### In silico analysis

Prediction of β-actin phosphorylation sites was performed using open-access NetPhos 3.1 Server (Center for Biological Sequence Analysis) (56). Predicted phosphorylation sites were confirmed by Western blot using the KinomeView Profiling kit antibodies. Prediction of the specific kinase for S239 phosphorylation of β-actin was performed using open-access NetPhosK Server (Center for Biological Sequence Analysis) (57) and Group-based Prediction System 3.0 (58).

### GEO analysis

Public array data analysis was performed in the R statistical environment (V4.1, R Core Team [2021]. R: a language and environment for statistical computing; R Foundation for Statistical Computing, Vienna, Austria, URL https://www.Rproject.org/). GEO data were accessed using the GEOquery package (59) and Array Express data were downloaded from https://www.ebi.ac.uk/arrayexpress/files/E-MEXP-3291/E-MEXP-3291.eSet.r. PLK1 expression was compared between sample groups by ANOVA in tidyverse (Alboukadel Kassambara [2020]. ggpubr: 'ggplot2′-based publication-ready plots. R package version 0.4.0. https://CRAN.Rproject.org/package=ggpubr) (60).

### Weighted gene co-expression network analysis

Analysis methods are described in detail in our recent article (22) and all data are freely available in the Mendeley dataset (61).

### Statistical analysis

Quantitative data were expressed as mean ± SEM. *t* test was performed for experiments with two treatment groups. All statistical analyses were carried out using Prism 7.0 (GraphPad).

## Data Availability

All data generated or analysed during this study are included in this article and its supplementary materials. Further enquiries can be directed to the corresponding author.

## Supplementary Material

Reviewer comments
