# Supplementary figures and images for: Polo-like kinase-1 mediates hepatitis C virus-induced cell migration, a drug target for liver cancer
Source: Life Sci Alliance. 2023 Aug 30;6(11):e202201630. doi: 10.26508/lsa.202201630 (PMC10468647; doi:10.26508/lsa.202201630)

Fig 2C

**Original blots**

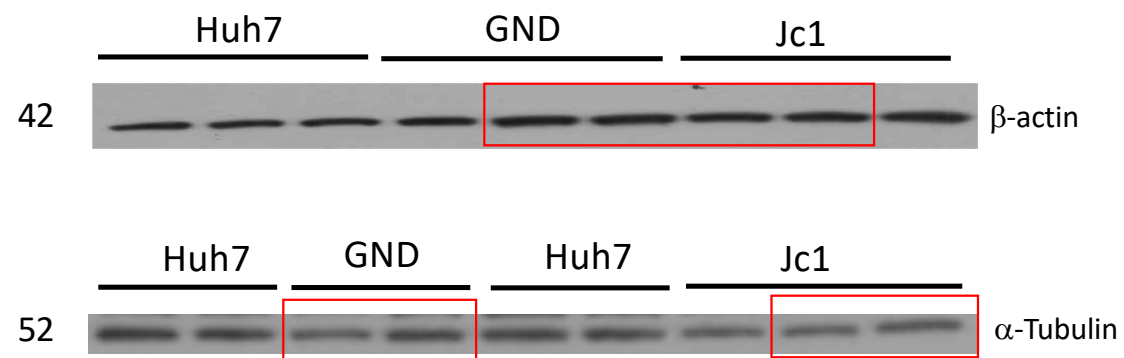

**Blots shown in Fig 2C**

**C**

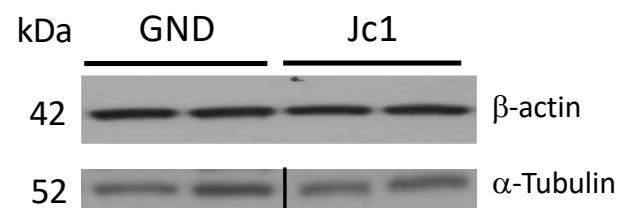

Supplement: Supplementary file 1 [file LSA-2022-01630_SdataF2.pdf]
