# Supplementary material for: Polo-like kinase-1 mediates hepatitis C virus-induced cell migration, a drug target for liver cancer
Source: Life Sci Alliance. 2023 Aug 30;6(11):e202201630. doi: 10.26508/lsa.202201630 (PMC10468647; doi:10.26508/lsa.202201630)
Supplement: Supplementary file 2 [file LSA-2022-01630_TableS1.docx]

**Supplementary Table 1**

| **Actin Isoforms** | **IHH** | | **WT** | | **S239D** | |
| --- | --- | --- | --- | --- | --- | --- |
|  | **Pellet** | **Supernatant** | **Pellet** | **Supernatant** | **Pellet** | **Supernatant** |
| **Total** |  |  |  |  |  |  |
| 200 x *g* | 43.59 | 56.41 | 72.76 | 27.24 | 64.47 | 35.53 |
| 1,500 x *g* | 40.90 | 59.10 | 31.66 | 68.34 | 29.31 | 70.69 |
| 16,000 x *g* | 49.54 | 50.47 | 37.41 | 62.59 | 53.86 | 46.14 |
| 66,000 x *g* | 67.56 | 32.45 | 57.20 | 42.80 | 74.42 | 25.58 |
| **β-actin** |  |  |  |  |  |  |
| 200 x *g* | 54.71 | 45.29 | 61.77 | 38.23 | 55.85 | 44.16 |
| 1,500 x *g* | 41.11 | 58.89 | 47.45 | 52.56 | 41.63 | 58.38 |
| 16,000 x *g* | 61.03 | 38.97 | 53.39 | 46.61 | 33.63 | 66.38 |
| 66,000 x *g* | 55.57 | 44.43 | 52.40 | 47.61 | 49.06 | 50.94 |
| **γ-actin** |  |  |  |  |  |  |
| 200 x *g* | 68.42 | 31.58 | 65.57 | 34.43 | 58.74 | 41.26 |
| 1,500 x *g* | 48.61 | 51.39 | 53.23 | 46.77 | 50.60 | 49.40 |
| 16,000 x *g* | 56.68 | 43.32 | 48.27 | 51.73 | 58.43 | 41.57 |
| 66,000 x *g* | 62.81 | 37.19 | 53.89 | 46.11 | 55.87 | 44.13 |
